# Body image differs in weight-based stereotypes between patients with bulimia nervosa and binge eating disorder: findings from the BodyTalk project

**DOI:** 10.1186/s40337-025-01201-5

**Published:** 2025-02-17

**Authors:** Adam Schweda, Paolo Meneguzzo, Jasmin Steinbach, Alexander Bäuerle, Maria Alejandra Quiros-Ramirez, Katrin E. Giel, Martin Teufel, Eva-Maria Skoda, Angela Favaro, Simone C. Behrens

**Affiliations:** 1 Clinic for Psychosomatic Medicine and Psychotherapy, University of Duisburg-Essen, LVR-University Hospital Essen, 45147 Essen, Germany; 2 Centre for Translational Neuro- and Behavioral Sciences (C-TNBS), University of Duisburg-Essen, 45147 Essen, Germany; 3 Neuroscience Department, University of Padova, Padua, Italy; 4 Padova Neuroscience Center, University of Padova, Padua, Italy; 5 Department of Perceiving Systems, Max Planck Institute for Intelligent Systems, Tübingen, Germany; 6 Department of Psychosomatic Medicine and Psychotherapy, Medical University Hospital Tübingen, University of Tübingen, Osianderstr. 5, 72076 Tübingen, Germany; 7 German Center for Mental Health (DZPG), Partner Site Tübingen, Tübingen, Germany; 8 Competence Center for Eating Disorders (KOMET), Tübingen, Germany; 9 Faculty of Electrical Engineering and Computer Science, Coburg University of Applied Sciences and Arts, Coburg, Germany

**Keywords:** Body image, Weight prejudice, Feeding and eating disorders, Self-concept

## Abstract

Bulimia Nervosa (BN) and Binge Eating Disorder (BED) have considerable symptom overlap, and prior studies observed similiarities in respect to patient’s body image. However, weight-based stereotypes have so far not been considered together with other facets of body image. This study investigated body image, body size estimation accuracy and weight-based stereotypes in a German-Italian sample of 22 patients with BN, 22 patients with BED and 44 weight-matched controls who had no eating disorder. Patients with BN and BED reported significantly more negative body image than controls and were not differently accurate in estimating their body size than the controls. However, patients with BN showed stronger weight-based stereotypes compared to patients with BED and controls, suggesting that these patients hold stronger stereotypes and apply weight-based stereotypes to lower weight excessively. Observations from this study suggest that clinical treatment of body image disturbance in patients with BN and BED should address negative body image and weight-based stereotypes in a differentiated way.

**Supplementary Information:**

The online version contains supplementary material available at 10.1186/s40337-025-01201-5.

## Abstract (plain language summary)

Both patients with bulimia nervosa and binge eating disorder have recurrent eating attacks and report issues with their body. At first glance, their symptoms occur very similar, however it is unclear whether their body image disturbance is actually as similar as it appears. After all, patients with binge eating but not those with bulimia nervosa are frequently overweight and visually fall out of the socially desired range. This study used sophisticated methodology to assess weight-based stereotypes held by patients along with body size estimation performance and body dissatisfaction. Results showed that the main difference between the two patient group’s body image is indeed in weight-based stereotypes. Patients with bulimia nervosa hold strong stereotypes already towards healthy weight whereas patients with binge eating do not differ from weight-matched controls.

**Supplementary Information:**

The online version contains supplementary material available at 10.1186/s40337-025-01201-5.

Bulimia Nervosa (BN) and Binge Eating Disorder (BED) are both characterized by recurrent binge eating episodes [12]. Diagnostic cross-over occurs in about 9% of the cases [5], and there is first evidence of shared genetic risk factors for both disorders [18]. Although it is a diagnostic criterion for BN only, patients with both diagnoses often report poor body image with high body dissatisfaction and overvaluation of weight and shape [22]. In both disorders, weight-based stereotypes play a role, defined as negative attitudes about overweight and obesity that can be the basis of weight stigma, i.e. social devaluation and mistreatment of overweight and obese people [38]. In BN, it has been suggested that poor body image might be driven by weight stigma, i.e. weight-based stereotypes that patients hold and that manifest in various cognitions and behaviors [1, 10, 26, 37], whereas the mechanisms remain unknown. Since body image disturbance is a known risk factor for onset, maintenance [1, 10, 37], a better understanding of weight-based stereotypes and its relation to body image disturbance in BED and BN could help to improve tailored care.

Although body image is conceptualized as a multifaceted construct that includes cognitive, affective, behavioral and perceptual aspects [20, 23], cognitive processes tap into all facets of body image. For example, it has been shown that in body size estimation tasks, usually seen as a measure of perceptual body image, overweight and obese individuals under- or overestimate their body size depending on exact task characteristics such as the visualization of their own identity [8, 46]. and precise task characteristics and cognitive biases influence performance [31–33]. Similary, affective reactions to the own body are modified by cognitions [47] and there are strong associations between behavioral and cognitive measures of body image [44]. Hence, to understand and treat body image disorder adequately, it is crucial to precisely characterize the underlying cognitive processes.

Assessment of affective and cognitive body image is usually conducted through a pool of validated body image questionnaires that assess evaluations different body regions as “okay” or “too big” [11] or preoccupation with and worry about one's body [9]. Normative cognitions such as weight-based stereotypes and weight stigma are not regularly assessed, but it has been shown that they [13, 25]. Assessing weight-based stereotypes is non-trivial, since stigmatizing is generally considered socially non-desirable [41]. However, a comprehensive assessment of weight-based stereotypes and how they relate to other facets of body image might support the development of interventions for body image disorder in BN and BED, either within a transdiagnostic or within a disorder-tailored approach.

The present study extends on existing work from the BodyTalk project and investigates differences between individuals with BED, BN and controls in cognitive, affective and perceptual body image, specifically in (1) self-reported body image, (2) body size estimation and (3) weight-based stereotypes. Among stigma-related variables, self-held weight-based stereotypes were chosen because they are the most basic cognitions related to weight stigma and can be better assessed indirectly than specific behaviors. We operationalized weight-based stereotypes via the valence of language associated with bodies of varying weight, following an approach using a machine-learned body shape model by Hill et al. [16] and Streuber et al. [45] that was adapted for the assessment of weight-based stereotypes within the BodyTalk project [3, 28, 30].

Based on the literature reviewed above, we hypothesized that individuals with BED would have a more negative body image than controls and exhibit an overestimation of their body size. Similarly, for individuals with BN, we expected a more negative body image than controls along with body size overestimation. Concerning weight-based stereotypes in individuals with BED, we hypothesized to find weight-based stereotypes against overweight or obese bodies in all groups. However, we explored whether indiviuals with BED to show stronger stereotypes than controls and hypothesized that indiviuals with BN might show stereotypes already towards bodies with normal weight.

## Methods

### Participants

Patients were recruited within the Body-Talk-project [3, 28, 29]. Participant assessment took place in two recruitment centers in Tübingen and Essen (Germany) and in Padova (Italy). Patients were recruited consecutively from outpatient departments and inpatient services. All study centers provide specialist services for eating disorders. Control participants were recruited from the local community in all three centers and screened for eating disorders prior to inclusion.

Overall, 22 patients with BN and 22 patients with BED were recruited. Only patients diagnosed with bulimia nervosa (ICD-10 F50.2), subsyndromal bulimia nervosa (ICD 10 F50.3), binge eating disorder or subsyndromal binge eating disorder (according to DSM-5: 307.51) were included into the respective groups. The control group was matched in terms of Body Mass Index (BMI; kg/m^2^) to control for effects of own body weight (BN vs non-overweight controls: W = 224, p = 0.70; BED vs. overweight controls: W = 262, p = 0.70). Hence, the control group consisted of 22 overweight and 22 non-overweight individuals.

Patients were considered eligible if they were adult, proficient in the German/Italian language, were able to give informed consent, did not suffer from neurological diseases, were not pregnant or lactating, not suicidal, and indicated to not suffer from psychotic, substance-related, bipolar, or body dysmorphic disorders. Furthermore, participants from the control group were *excluded* if they reported a current or lifetime diagnosis of a mental disorder. They were not allowed to be engaged in current attempts to lose weight (e.g. dieting behavior or restricted eating behavior). To ensure adequacy, participants were screened in a brief interview, completed the EDE-Q [15], and were excluded if the latter indicated potentially disturbed eating behavior. All participants provided written informed consent to participate in the study. The study was approved by the local ethics committees.

### Study materials and procedure

All participants completed a single session of approximately 30 min in which they conducted a set of computerized tasks (rating task and adjustment task, see below for details) and completed a set of self-report questionnaires. Instruments assessing patient characteristics were the depression module of the Patient Health Questionnaire (PHQ-9; [19]), the Rosenberg self-esteem scale, (R-SES; [49]), and an inhouse-questionnaire including sociodemographic, medical and anthropometric information such as age, height, weight, lowest weight ever, age at first diagnosis, and duration of current symptoms. Weight bias was assessed using the Fat Phobia Scale (FPS; [2]). Body image was assessed using the Eating Disorder Examination Questionnaire (EDE-Q; [9]), the Body Image Questionnaire (FKB-20; [6]) as well as the Physical Appearance Comparison Scale (PACS; [31, 32]) in German respectively Italien versions.

#### Rating task

For the rating task, we used, according to biological sex of the participant, a selection of 12 female or male bodies selected from the Streuber et al. [45] dataset. The body shapes in this dataset were generated using a statistical model of 3D human body shape (SMPL; [24]). Within this model, 3D body shape is represented based on principal components. The bodies used in these studies were generated by sampling values of the first eight shape defining principle components following a normal distribution. As a result, bodies in this dataset represent random body shapes whose BMI were estimated as following: height (in meters) was determined by subtracting the lowest point of the body mesh from its highest point. Weight of the mesh in kilograms was obtained by calculating the volume and dividing it by the human body density (on average 1010 kg/m^3^). The selected bodies had a BMI from 15.5 to 36.5 kg/m^2^. The body shapes varied randomly within anthropometrically plausible shapes within defined weight categories; three avatars each fell into in the underweight, normal weight, overweight, and obese range. The bodies were fed into a computerized task, in which each of the 12 bodies was presented 16 times in a row along with each one of 16 character or shape describing adjectives. Order of bodies and adjectives were randomized. Adjectives were selected from a set of adjectives that described body shapes (e.g. “big”, “apple-shaped”, “feminine”), as they were used in Streuber et al. [45] as well as prior literature on weight stigma [14, 39]. Equivalence of the two langage versions was discussed and agreed by two native speakers. In each of the 192 trials, participants rated how well the adjective matched the displayed body on a 4-point scale from “very much” to “not at all”. The participants were instructed to decide spontaneously, and it was explicitly stressed that there was no right or wrong. Completion of the rating task took about 10 min.

#### Adjustment task

In order to assess body shapes that participants specifically associate with specific language, we adapted and used a tool that was initially described in Streuber et al. [45]. In this task, participants were asked to generate prototypic bodies that matched the 16 adjectives used in the rating task. After an initial test trial, participants were presented adjectives from the rating task in random order. They were granted unlimited time to construct a 3D-avatar, using eight sliders that each manipulated a shape forming principal component. In the same vein, participants were prompted to generate a body that subjectively best matched their own body. Completion of the adjustment task took about 8 min.

#### Valence assessment

In order to assess the emotional valence of the words used in the rating task and the adjustment task, participants were asked to rate each of the 16 adjectives on a 5-point scale ranging from “clearly negative” to “clearly positive”. Here, the adjective was simply presented on a screen. The order of presentation was pseudo-randomized. Completion of this task took about 2 min.

### Data analysis

#### Sample characteristics and body image

To analyze similarities and differences between the groups in sample characteristics, several between-subject ANOVAs with Tukey-corrected post-hoc tests were conducted. For categorical variables, χ^2^-Tests or Fisher’s tests were applied depending on the minimum cell size.

#### Body size estimation

Performance in body size estimation was operationalized using the body perception index (BPI; [43]). Here, the BMI of the shaped avatar was derived according to the formula already used on the mesh dataset. Subsequently, the resulting BMI was divided by the participants’ actual BMI (c.f. [3]) and multiplied by 100. Participants with BPIs of < 100 underestimate their weight, and participants with a BPI > 100 overestimate their weight. ANOVAs were used to test whether there were group-wise differences.

#### Weight-based stereotypes

Self-reported weight-based stereotypes on the fat phobia scale was analyzed using ANOVA with Tukey-corrected post-hoc tests. For analysis of the rating task, generalized estimated equations (GEE) were used to regress the BMI of the presented 3D-avatars, the group (BN vs. BED vs. healthy controls), as well as the individual valence ratings of the presented adjectives on the ratings of the bodies. In general, GEE models are an alternative to mixed linear models when subject- or cluster-wise effects (i.e. random effects) are not central to the hypothesis, but a correction for intra-cluster correlations is still necessary (see e.g. [27]). Furthermore, sandwich estimators were applied to standard errors, which ensures more robustness against violations of homoscedasticity. Compared to mixed linear models, the GEE-approach does not require normality of random effects. We generally applied an “exchangeable” covariance structure which assumes the same correlation across measurements. This was due to the lack of any prior assumption about differential within-subject measurement correlations—contrary to e.g. time-series data. Apart from regression weights, variable-wise χ^2^-tests are provided in our results section to better illustrate global between-group differences. To test group differences, simple slope and simple effect analyses were used to explore differential relationships between rating and BMI for each valence between the groups.

To control for group differences in valence ratings, we investigated whether the valence ratings for the applied adjectives differ between groups using a mixed ANOVA, regressing the respective adjectives (as a repeated measures factor) and group (as a between-subject factor) on the respective valence rating. If, for instance, participants with bulimia nervosa systematically rated negative words more negatively, there should be a significant interaction effect between these two factors.

In the adjustment task, the constructed 3D-avatars’ final BMIs were used as the outcome variable. Here, another GEE model with an exchangeable covariance structure was set up to test for group differences, including *group* (BN vs. BED vs. healthy controls) and *valence* of the presented attribute as predictor variables. This way, differences in the constructed avatars’ shape based on valences of presented adjectives were captured.

For the sake of conciseness, results are presented with one aggregated control condition, including overweight and non-overweight control participants. Yet, all central analyses were reproduced with two control groups to rule out eventual effects of BMI. These are presented in detail in the supplemental online material.

#### Software

All analyses were performed using R 3.6.3 [40]. For analyses of variance (ANOVA), the afex package was used [42]. GEE models were performed using geepack [17]. Marginal effects analyses were carried out using the package emmeans [21].

## Results

### Sample characteristics

Each 1 participant from the BN and BED group indicated that their sex was male, and so did 3 participants from the control group. An overview of the sample characteristics, as well as comparisons between the three groups is provided in Table 1. Overall, both BN and BED had higher depression scores according to PHQ-9, lower self-esteem according to R-SES and reported more restrained eating, eating concern and overall eating disorder symptoms (EDE-Q) than the control group, each with large effect sizes. The BN and BED group did not differ regarding the above variables. BMI was lowest in the BN group and highest in the BED group. Note that the control group was weight matched for both patient groups such that it spanned a large range of BMI and was, on average, overweight (see supplemental online material for further analyses). There were no significant differences between German and Italian participants (see supplemental material for detailed analyses).

**Table 1:** Participant characteristics, body image and body size estimation performance of the different groups

	BN (n = 22)	BED (n = 22)	Controls (n = 44)	p	Effect size (ƞ^2^)
	M (SD)	M (SD)	M (SD)		
Age at diagnosis (years)	20 (7.31)^b^	26.5 (11.6)^a^		0.046	0.11
Age (years)	26.7 (9.71)	34.7 (13.2)	32.3 (13.7)	0.101	0.05
body mass index (kg/m^2^)	21.20 (2.67^b,c^	37.86 (8.63)^a,c^	29.51 (11.48)^a,b^	< 0.0.001	0.29
Body perception index (BPI)	106.90 (14.29)^b,c^	91.55 (17.65)^a^	94.88 (18.70)^a^	0.009	0.11
EDE-Q—restraint (1–6)	2.59 (1.64)^c^	2.48 (1.71)^c^	1.32 (1.23)^a,b^	0.001	0.15
EDE-Q—eating concern (1–6)	3.16 (1.54)^c^	2.57 (1.73)^c^	0.35 (0.58)^a,b^	< 0.001	0.53
EDE-Q—weight concern (1–6)	3.25 (1.98)^c^	4.00 (1.02)^c^	1.55 (1.28)^a,b^	< 0.001	0.37
EDE-Q—shape concern (1–6)	3.94 (1.75)^c^	4.74 (1.16)^c^	1.86 (1.53)^a,b^	< 0.001	0.42
EDE-Q—total score (1–6)	3.24 (1.52)^c^	3.45 (1.14)^c^	1.27 (1.00)^a,b^	< 0.001	0.45
PHQ-9—depression score	12.82 (5.76)^c^	14.41 (5.55)^c^	4.57 (2.58)^a,b^	< 0.001	0.53
FPS—fat phobia scale	49.23 (6.58)	49.14 (7.59)	49.07 (5.24)	0.995	< 0.001
FKB-20—perception of body dynamics	29.23 (5.78)^c^	24.79 (7.33)^c^	35.09 (6.69)^a,b^	< 0.001	0.31
FKB-20—negative body image	35.32 (8.58)^c^	39.27 (6.51)^c^	26.14 (10.34)^a,b^	< 0.001	0.29
R-SES—self-esteem	15.59 (4.70)^c^	14.57 (5.51)^c^	21.55 (5.04)^a,b^	< 0.0.001	0.30
PACS—physical appearance comparison Scale	17.41 (3.90)^c^	18.95 (2.94)^c^	15.13 (3.17)^a,b^	< 0.001	0.20

EDE-Q: Eating Disorder Examination Questionnaire [15]; PHQ-9: Patient Health Questionnaire [19], FPS: Fat Phobia Scale [2], FKB-20: Body Image Questionnaire [6], R-SES: Rosenberg Self-Esteem Scale [49]; PACS: Physical Appearance Comparison Scale [31, 32]. Uppercase letters represent results of Tukey-corrected post-hoc comparisons (^a^significantly different from the BN-group, ^b^significantly different from the BED-group, ^c^significantly different from control group)

### Body image

An overview of body image parameters is provided in Table 1. As we hypothsized, the BN and BED group reported a more negative body image and stronger habits in comparing their physical appearance than the control group with no differences between the two patient groups and large effect sizes.

### Body size estimation

The univariate ANOVA comparing the BPI between all three groups yielded significant differences between the three groups (F(2, 84) = 4.93, p = 0.009, η^2^ = 0.11; bulimia nervosa vs. binge eating: estimate = 0.15, t(84) = 2.98, p = 0.01; bulimia nervosa vs. healthy controls: estimate = 0.12, t(84) = 2.63, p = 0.03). A comparison of the group-wise means of the BPI revealed that patients with BN slightly overestimated their body weight toward a higher BMI, while the BED group and healthy controls underestimated their own body weight. Yet, these effects were driven by the participants’ BMI: Supplemental analyses revealed that patients with BN, as well as non-obese control participants tend to slightly overestimate their body shape, while patients with BED and obese control participants slightly underestimated it (see supplementary materials for details). There was no effect that can be specifically attributed to the presence of an eating disorder. Hence, our hypothesis that patients would overestimate their size compared to controls was not confirmed.

### Weight-based stereotypes

There was no significant difference in self-reported weight-based stereotypes on the Fat Phobia Scale between the groups (*cf*. Table 1).

#### Differences in valence ratings of the adjectives

To check for a priori differences between groups in terms of valence perception, a mixed ANOVA with the between factor *group* (BN vs. BED vs healthy controls) and the repeated measures factor *adjective* was performed. The interaction effect would indicate a differential rating of positive, negative or neutral words for any of the groups. Yet, such effect did not reach statistical significance (group x adjective interaction: F(14.03, 596.21) = 1.09, p = 0.37, η^2^ = 0.02). There was a significant main effect of group (F(2, 85) = 3.63, p = 0.031, η^2^ = 0.01), pointing towards a small difference in overall response tendencies of the groups, which is not specific to any of the stimuli but refers to all of them (see [7], and supplementary materials). As hypothesized, Tukey-corrected post hoc tests revealed that patients with BN tend to be more reserved with positive valence ratings in their responses compared to the healthy controls (estimate =  − 0.169, t(85) =  − 2.631, p = 0.027). As intended when designing the task, there was also a significant main effect of *adjective* (F(14.03, 596.21) = 209.03, p < 0.001, η^2^ = 0.69) on valence ratings. As expected, adjectives such as open-minded or feminine were generally rated more positively than adjectives like lazy or impulsive (all p < 0.001, see supplementary materials for an illustration).

#### Rating task

**Fig. 1:**
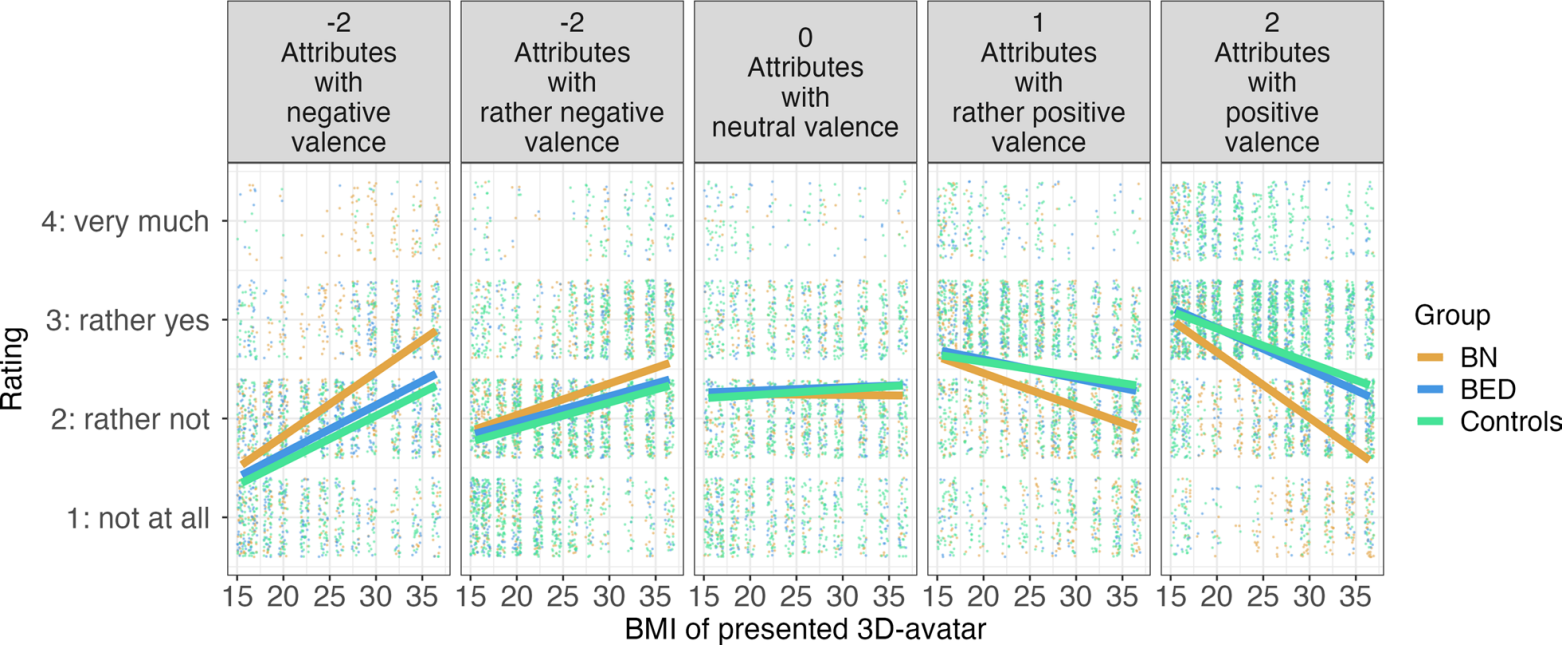
Results from the rating task. Ratings indicate how much the adjective matched the displayed body. Participants in the BN group assigned significantly higher fit with negative adjectives and less with positive adjectives when they rated avatars with higher BMIs compared to patients with BED and healthy controls (see left- and rightmost panels). The lines represent marginal effects of the GEE, and points represent the raw data (in a jittered fashion)

For the rating task, the Generalized Estimated Equations (GEE) model yielded significant effects of valence (χ^2^(1) = 436, p < 0.001), and of the two-way interactions between valence and BMI (valence X BMI: χ^2^(1) = 482, p < 0.001), as well as between group and valence (group X valence: χ^2^(2) = 90, p < 0.001). Importantly, the three-way interaction between BMI, valence and group reached significance with χ^2^(2) = 24, p < 0.001). As hypothesized, Tukey-corrected simple slope analyses revealed that for participants with BN, the relationship between the BMI and the rating is described by a significantly steeper negative linear slope than in the BED and healthy control groups at a valence rating of 2, which is positive (BN vs. BED: estimate =  − 0.02, z =  − 3.54, p = 0.001; BN vs. healthy controls: estimate =  − 0.03, z =  − 5.41, p < 0.001, see Fig. 1). No such difference occured between the BED and the control group (estimate =  − 0.007, z =  − 1.181, p = 0.464). Further simple effect analyses, comparing the predicted means at different levels of BMI at a valence of 2 showed that regression lines start to diverge early at BMIs of about 22.3 kg/m^2^ (see supplemental online material). In other words, participants in the BN group assigned adjectives with maximally positive ratings less frequently to medium to high BMI than patients with BED and healthy controls. This divergence was even more pronounced at higher BMIs. A similar, yet less distinct effect were observed for attributes with prior valence ratings of 1 (rather positive) on a scale of − 2 to 2. Slopes differed significantly between the BN and both other groups (BN vs. BED: estimate =  − 0.01, z =  − 2.84, p = 0.01; BN vs. healthy controls: estimate =  − 0.02, z =  − 4.49, p < 0.001, see Fig. 1). The divergence between BN and the other groups emerged at a BMI of 20 kg/m^2^ (see supplemental online material for a summary all marginal effects). Hence, patients with BN assigned adjectives with a valence rating of 1 (rather positive) less frequently to medium or heavy weighted avatars compared to participants with BED and healthy control participants. No differences in slopes occured at valences of 0 (completely neutral) and − 1 (rather negative, all p > 0.20). Yet, group differences reemerged for attributes with a valence of − 2 (negative): Here, patients with BN showed a steeper positive slope than healthy controls (estimate = 0.02, z = 2.51, p = 0.03, see Fig. 1), suggesting that they assigned these attributes readily to bodies of medium to high BMI. The comparison between BN and BED yielded a p-value of p = 0.01 (estimate = 0.016, z = 1.97). Simple effect analyses on each of presented avatars’ BMI revealed that differences emerge at low BMIs of 20.0 on and become larger at high BMIs. Differences between patients with BN and patients with BED were significant (see supplemental online material for a comprehensive analysis). Overall, participants with bulimia nervosa tended to assign less positively valent and more negatively valent attributes to higher BMIs than healthy controls, and more negatively valent attributes to higher BMIs than patients with BED. A similar pattern emerged when the control group was disaggregated into an obese and a non-obese group (see supplemental online material). Comparing the participants from the two countries, the results from the German subsample appear almost identical to the aggregated ones, but an analysis of the Italian subsample yields some indications that participants in the BED group tended to be more reserved in rating high-BMI avatars positively (see supplemental online material).

#### Adjustment task

**Fig. 2:**
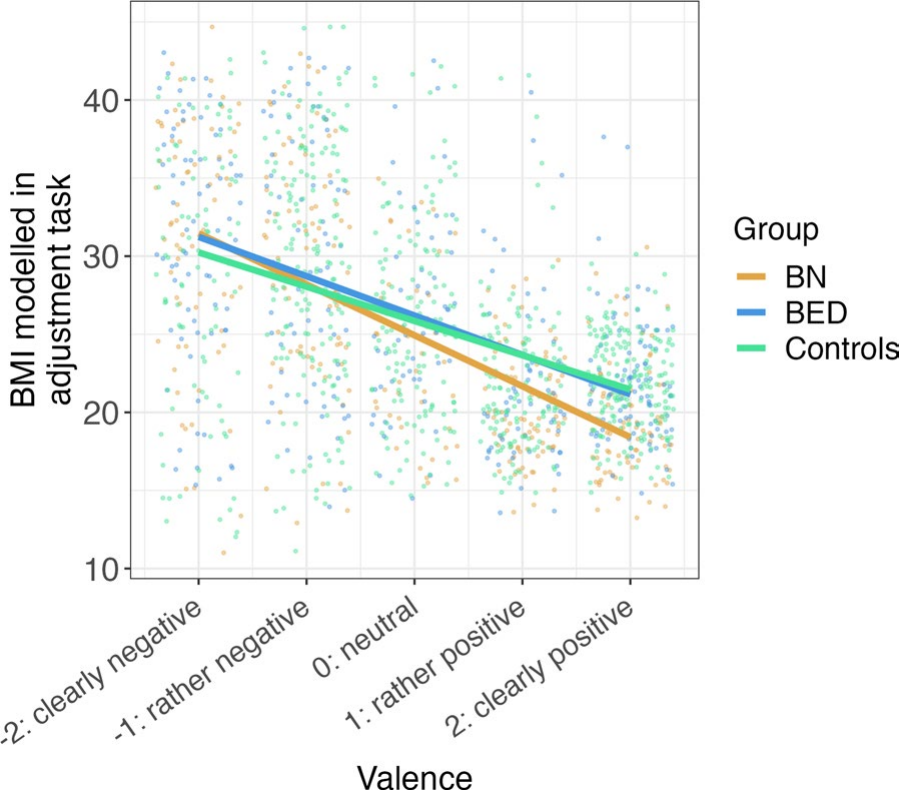
BMI of adjusted bodies based on valence of the target attribute. In the adjustment task, participants generated 3D-avatars to match a prototype for the above listed adjectives. The y-axis depicts the generated avatars’ BMI, and the x-Axis the valence of the respective attribute. Lines are based on marginal effects of the GEE-model. The overlaid jitter plot displays raw data points. The BN group constructed avatars with lower BMIs based on attributes that are rated more positively.

Another GEE model was used to assess if BMI of prototypic 3D-avatars that participants generated for adjectives of different valence differed between the BN, BED and control groups. The factorial group variable, the valence of the presented attribute, as well as the interaction of both these terms were regressed on the BMI of the generated avatar. Clusters for each participant and each attribute were added. We specified an “exchangeable” working covariance for our model. χ^2^-tests for both variables—group and valence—show that there are either differences between groups, or a slope that significantly differs from zero (valence: χ^2^(1) = 487, p < 0.001; group: χ^2^(2) = 10, p < 0.01). Although lower order predictors should be interpreted with caution in case of a present interaction, Fig. 2 clearly shows that higher valence of the modeled adjective goes along with a less voluminous body shape modeled. Importantly though, we observed a significant interaction between group and valence (χ^2^(2) = 15, p = 0.001). Subsequent simple slope analyses of the relationship between valence of the attribute used to create the 3D-avatar and the BMI of the 3D-avatar revealed a steeper negative regression line for the BN group compared to the BED (estimate =  − 0.74, z = 2.39, p = 0.04) and the control group (estimate =  − 1.07, z =  − 3.84, p < 0.001, see Fig. 2). Simple effect comparisons show that for attributes that were rated with valences of 1 (rather positive) and 2 (positive), patients with BN generate avatars with significantly lower BMIs than patients with BED and healthy controls (all p < 0.001). Such effect is also partly visible at a valence of 0 (neutral valence; BN vs BED: estimate =  − 1.26, z =  − 2.68, p = 02; BN vs healthy controls =  − 0.92, z =  − 2.26, p = 0.06). Participants with BN, hence, construct avatars with more thin body shapes based on positive or neutral attributes. An analysis with a disaggregated control group yielded similar results (see supplemental online material).

## Discussion

In this study, we observed that patients with BED and BN both report negative body image yet differ in weight-based stereotypes. Specifically, patients with BN showed stronger weight-based stereotypes in the sense that negatives attributes were more strongly associated to higher body weight compared to patients with BED and controls, and applied already towards lean healthy weight. Differences in body size estimation accuracy can be attributed to participant’s BMI rather than presence of an eating disorder. Both patients with BN and BED have a more negative body image than BMI-matched controls. Our observations suggest that despite of clinical similarities in negative body image, BN and BED have distinct underlying mechanisms in cognitive and affective body image facets, which should be considered in interpreting body image data and targeted in individualized treatment regimes.

In our study, we examined a German-Italian sample of patients with BN and BED that was compared to a weight-matched control sample. Both samples were recruited consecutively from clinical routine and their self-report of eating disorder symptoms and body image matches norm values reported in the literature [15]. Regarding the negative self-reported body image, our observations are consistent with previous literature [22] in that both patient groups had more pathological scores than the weight-matched control group but did not differ significantly from each other. However, it needs to be acknowledged that due to our recruitment within specialist centers adhering to university hospitals, we might have assessed a selection of severe cases of BN and BED compared to a mere outpatient sample.

While participants did not report any weight-based stereotypes in the Fat Phobia Scale, we clearly observed weight-based stereotypes in our computerized tasks, i.e. all participants associated higher body weight with negative attributes and lower body weight with positive attributes. This observation is in line with previous literature suggesting that although few people endorse holding weight-based stereotypes, the majority still reports weight-based stereotypes when asked more indirectly [35]. Weight-based stereotypes were observed in all groups, including both individuals with BED and controls with overweight. This observation emphasizes that lived experience with overweight is not protective for weight-based stereotypes but in contrast an integrative part of body-related cognitions throughout the BMI range [34, 50]. Since we did not observe this effect to be specific for patients with BED, we suspect weight-based stereotypes to be a factor for negative body image independent from specific symptoms of BED. For clinical management of BED, targeting both weight-based stereotypes and negative body image could still be a promising strategy [36].

Comparing individuals with BN and BED, weight-based stereotypes were significantly more pronounced in individuals with BN. Specifically, this group assigned more positive attributes to lean bodies than all other groups, which suggests overvaluation of low body weight which is not present in controls and individuals with BED. Further, patients with BN assigned negative attributes to high body weight starting from a lower BMI than all other groups. While previous studies on patients with anorexia nervosa [3, 33, 48] suggested that standards overvaluing a thin *own* body are key to body image disturbance, our observations suggest a different mechanism for individuals with BN. Voges et al. [48] report that patients with BN apply less double standards in judging body sizes than patients with anorexia nervosa. In line with this observation, our data suggests that individuals with BN hold strong negative weight-bases stereotypes towards bodies *in general* which are already applied to normal weight. Hence, extreme weight-based stereotypes could be a mechanism that drives social comparisons [4] and thus body dissatisfaction and drive for thinness in patients with BN.

For body size estimation, we observed that irrespective of diagnoses, participants with normal weight slightly overestimated their body size whereas participants with overweight slightly underestimated their body size. This finding does not match our hypothesis that patients might exaggerate their own weight category, but it is generally in line with previous literature that assessed body size estimation with depictive tasks [31, 32]. Our observation that BMI and not presence of an eating disorder diagnosis predicted size estimation accuracy is indicative of general perceptual biases (e.g., contraction bias; [8]) rather than a disorder specific symptom. Of note, the over- and underestimations of our participants were in the opposite direction than reported for tasks referring to the own identity [46]. Hence, our intention to generate self-reference through the instruction of generating the own body was probably not sufficient to actually induce biases as they have been observed for the own identity. Additionally, the task order with body size estimation at the end might have made weight-based stereotypes more salient and participants biased their size estimation towards a positive rated body. Moreover, it could have been a method-specific effect because participants had low experience with viewing and adapting their body as a 3d avatar.

Although we consider our experimental approach superior to self-report in assessing weight-based stereotypes, our tasks nevertheless bear limitations. While these tasks produced face valid results in several samples [3, 28], they still present average body shapes with grey shaded textures. For size estimation, prior studies have shown that stimuli without accurate identity may bias the performance [31, 32, 46]. Although we assessed weight-based stereotypes more indirectly than self-report of stereotypes in a questionnaire, it cannot be ruled out that participants guessed the focus of our task and attempted to adjust their responses to a socially accepted level, thus underestimating existing weight-based stereotypes. Furthermore we only assessed overall weight-based stereotype, but not how much participants adopted these stereotypes for the self-esteem, which could have been helpful in the clinical interpretation of our observations. Finally, we performed the task at three study centers in two different countries (Germany and Italy). While this might prima facie increase generalizability, we encountered some inconsistencies while reanalyzing the data taking the respective subsample into consideration that are detailed in the supplemental online material. This underlines the need for replications with higher sample sizes, despite a relatively high statistical power (see supplemental material).

Overall, our study contributes to the understanding of similarities and differences of body image in patients with BN and BED, and, thus, may add into debates about unified diagnostic criteria and treatment optimization. Our study emphasizes that despite phenomenological similarities, there are differences in body image between the two disorders. We therefore advocate for more detailed studies and for the development of treatment modules that address negative body image and question weight-based stereotypes in a differentiated way.

## Supplementary Information

Supplementary Material

## Data Availability

Due to a current guideline of the University Hospital Tübingen data protection team, data are considered as sensitive and cannot be publicly provided. Interested authors are invited to contact the corresponding author to find individual solutions for data sharing.

## References

[1] Askew AJ, Peterson CB, Crow SJ, Mitchell JE, Halmi KA, Agras WS, et al. Not all body image constructs are created equal: predicting eating disorder outcomes from preoccupation, dissatisfaction, and overvaluation. Int J Eat Disord. 2020;53(6):954–63. 32304257 10.1002/eat.23277 PMC9382219

[2] Bacon JG, Scheltema KE, Robinson BE. Fat phobia scale revisited: the short form. Int J Obes. 2001;25:252–7. 10.1038/sj.ijo.0801537 11410828

[3] Behrens SC, Meneguzzo P, Favaro A, Teufel M, Skoda EM, Lindner M, et al. Weight bias and linguistic body representation in anorexia nervosa: findings from the BodyTalk project. Eur Eat Disord Rev. 2021;29(2):204–15. 33252835 10.1002/erv.2812

[4] Blechert J, Nickert T, Caffier D, Tuschen-Caffier B. Social comparison and its relation to body dissatisfaction in bulimia nervosa: evidence from eye movements. Psychosom Med. 2009;71(8):907–12. 19661192 10.1097/PSY.0b013e3181b4434d

[5] Castellini G, Lo Sauro C, Mannucci E, Ravaldi C, Rotella CM, Faravelli C, et al. Diagnostic crossover and outcome predictors in eating disorders according to DSM-IV and DSM-V proposed criteria: a 6-year follow-up study. Psychosom Med. 2011;73(3):270–9. 21257978 10.1097/PSY.0b013e31820a1838

[6] Clement U, Löwe B. Body image questionnaire (BIQ-20), manual with questionnaire and assessment tools. Göttingen: Hogrefe; 1996.

[7] Cohen J. Statistical power analysis for the behavioral sciences. 2nd ed. Hillsdale: Erlbaum; 1988.

[8] Cornelissen KK, Gledhill LJ, Cornelissen PL, Tovee MJ. Visual biases in judging body weight. Br J Health Psychol. 2016;21(3):555–69. 26857215 10.1111/bjhp.12185

[9] Fairburn CG, Beglin SJ. The assessment of eating disorders: interview or self-report questionnaire? Int J Eat Disord. 1994;16:363–70. 7866415

[10] Fairburn CG, Cooper Z, Shafran R. Cognitive behaviour therapy for eating disorders: a “transdiagnostic” theory and treatment. Behav Res Ther. 2003;41(5):509–28. 12711261 10.1016/s0005-7967(02)00088-8

[11] Garner DM, Olmstead MA, Polivy J. Development and validation of a multidimensional eating disorder inventory for anorexia nervosa and bulimia. Int J Eat Disord. 1983;2(2):15–34.

[12] Giel KE, Bulik CM, Fernandez-Aranda F, Hay P, Keski-Rahkonen A, Schag K, et al. Binge eating disorder. Nat Rev Dis Prim. 2022;8(1):16. 35301358 10.1038/s41572-022-00344-y PMC9793802

[13] Griffiths S, Mitchison D, Murray SB, Mond JM, Bastian BB. How might eating disorders stigmatization worsen eating disorders symptom severity? Evaluation of a stigma internalization model. Int J Eat Disord. 2018;51(8):1010–4. 30055009 10.1002/eat.22932

[14] Hellbardt M, Riedel-Heller SG, Sikorski C. Dietitians’ attitudes towards obese patients. Ernährungs Umschau. 2014;61(5):78–81.

[15] Hilbert A, Tuschen-Caffier B, Karwautz A, Niederhofer H, Munsch S. Eating disorder examination-questionnaire. Diagnostica. 2007;53(3):144–54.

[16] Hill MQ, Streuber S, Hahn CA, Black MJ, O’Toole AJ. Creating body shapes from verbal descriptions by linking similarity spaces. Psychol Sci. 2016;27(11):1486–97. 27708127 10.1177/0956797616663878

[17] Højsgaard S, Halekoh U, Yan J. The R package geepack for generalized estimating equations. J Stat Softw. 2006;15(2):1–11.

[18] Hubel C, Abdulkadir M, Herle M, Loos RJF, Breen G, Bulik CM, et al. One size does not fit all. Genomics differentiates among anorexia nervosa, bulimia nervosa, and binge-eating disorder. Int J Eat Disord. 2021;54(5):785–93. 33644868 10.1002/eat.23481 PMC8436760

[19] Kroenke K, Spitzer RL. The PHQ-9: a new depression diagnostic and severity measure. Psychiatric Ann. 2002;32(9):509–15.

[20] Legenbauer T, Vocks S, Betz S, Báguena Puigcerver MJ, Benecke A, Troje NF, et al. Differences in the nature of body image disturbances between female obese individuals with versus without a comorbid binge eating disorder: an exploratory study including static and dynamic aspects of body image. Behav Modif. 2011;35(2):162–86. 21324945 10.1177/0145445510393478

[21] Lenth R. emmeans: estimated marginal means, aka least-squares means (Version 1.8.2); 2022

[22] Lewer M, Bauer A, Hartmann AS, Vocks S. Different facets of body image disturbance in binge eating disorder: a review. Nutrients. 2017;9(12):1294. 29182531 10.3390/nu9121294 PMC5748745

[23] Longo MR. Implicit and explicit body representations. Eur Psychol. 2015;20(1):6–15.

[24] Loper M, Mahmoud N, Romero J, Pons-Moll G, Black MJ. SMPL: a skinned multi-person linear model. ACM Trans Graph. 2015;34(248):1–16.

[25] Marshall RD, Latner JD, Masuda A. Internalized weight bias and disordered eating: the mediating role of body image avoidance and drive for thinness. Front Psychol. 2019;10:2999. 32038383 10.3389/fpsyg.2019.02999 PMC6987958

[26] McEntee ML, Philip SR, Phelan SM. Dismantling weight stigma in eating disorder treatment: next steps for the field. Front Psychiatry. 2023;14:1157594. 37113547 10.3389/fpsyt.2023.1157594 PMC10126256

[27] McNeish D, Stapleton LM, Silverman RD. On the unnecessary ubiquity of hierarchical linear modeling. Psychol Methods. 2017;22(1):114–40. 10.1037/met0000078. 27149401 10.1037/met0000078

[28] Meneguzzo P, Behrens SC, Favaro A, Tenconi E, Vindigni V, Teufel M, et al. Body image disturbances and weight bias after obesity surgery: semantic and visual evaluation in a controlled study, findings from the bodytalk project. Obes Surg. 2021a;31:1625–34. 33405179 10.1007/s11695-020-05166-z PMC8012323

[29] Meneguzzo P, Behrens SC, Pavan C, Toffanin T, Quiros-Ramirez MA, Black MJ, et al. Exploring weight bias and negative self-evaluation in patients with mood disorders: insights from the BodyTalk Project. Front Psychiatry. 2024;15:1407474. 38873536 10.3389/fpsyt.2024.1407474 PMC11169709

[30] Meneguzzo P, Collantoni E, Bonello E, Vergine M, Behrens SC, Tenconi E, et al. The role of sexual orientation in the relationships between body perception, body weight dissatisfaction, physical comparison, and eating psychopathology in the cisgender population. Eat Weight Disord. 2021b;26:1985–2000. 33090374 10.1007/s40519-020-01047-7 PMC8292238

[31] Mölbert SC, Hautzinger M, Karnath HO, Zipfel S, Giel K. Validation of the physical appearance comparison scale (PACS) in a German sample: psychometric properties and association with eating behavior, body image and self-esteem. Psychother Psychosom Med Psychol. 2017a;67(2):91–7. 28288499 10.1055/s-0042-123842

[32] Mölbert SC, Klein L, Thaler A, Mohler BJ, Brozzo C, Martus P, et al. Depictive and metric body size estimation in anorexia nervosa and bulimia nervosa: a systematic review and meta-analysis. Clin Psychol Rev. 2017b;57:21–31. 28818670 10.1016/j.cpr.2017.08.005

[33] Mölbert SC, Thaler A, Mohler BJ, Streuber S, Romero J, Black MJ, et al. Assessing body image in anorexia nervosa using biometric self-avatars in virtual reality: attitudinal components rather than visual body size estimation are distorted. Psychol Med. 2018;48(4):642–53. 28745268 10.1017/S0033291717002008 PMC5964466

[34] Pearl RL, Puhl RM. Measuring internalized weight attitudes across body weight categories: validation of the modified weight bias internalization scale. Body Image. 2014;11(1):89–92. 24100004 10.1016/j.bodyim.2013.09.005

[35] Pearl RL, Wadden TA, Groshon LC, Fitterman-Harris HF, Bach C, LaFata EM. Refining the conceptualization and assessment of internalized weight stigma: a mixed methods approach. Body Image. 2023;44:93–102. 36549092 10.1016/j.bodyim.2022.12.002

[36] Pearl RL, Walton K, Allison KC, Troniert JS, Wadden TA. Preference for people-first language among patients seeking bariatric surgery. JAMA Surg. 2018;153(12):1160–2. 30193347 10.1001/jamasurg.2018.2702 PMC6583679

[37] Prnjak K, Hay P, Mond J, Bussey K, Trompeter N, Lonergan A, et al. The distinct role of body image aspects in predicting eating disorder onset in adolescents after one year. J Abnorm Psychol. 2021;130(3):236–47. 33705157 10.1037/abn0000537

[38] Puhl RM, Himmelstein MS, Pearl RL. Weight stigma as a psychosocial contributor to obesity. Am Psychol. 2020;75(2):274–89. 32053000 10.1037/amp0000538

[39] Puhl RM, Suh Y. Stigma and eating and weight disorders. Curr Psychiatry Rep. 2015;17(3):552. 25652251 10.1007/s11920-015-0552-6

[40] R Core Team. R: A language and environment for statistical computing. R Foundation for Statistical Computing, Vienna, Austria. 2021. https://www.R-project.org/.

[41] Ruggs EN, King EB, Hebl M, Fitzsimmons M. Assessment of weight stigma. Obes Facts. 2010;3(1):60–9. 20215796 10.1159/000273208 PMC6452119

[42] Singman H, Bolker B, Westfall J, Aust F, Ben-Shachar M. afex: analysis of factorial experiments (Version 1.1–1); 2022

[43] Slade PD, Russell GFM. Awareness of body dimensions in anorexia nervosa: cross-sectional and longitudinal studies. Psychol Med. 1973;3:188–99. 4715850 10.1017/s0033291700048510

[44] Steinfeld B, Bauer A, Waldorf M, Hartmann AS, Vocks S. Diagnostik der Körperbildstörung. Psychotherapeut. 2017;62(3):164–82.

[45] Streuber S, Quiros-Ramirez MA, Hill MQ, Hahn CA, Zuffi S, O’Toole A, et al. Body talk: crowdshaping realistic 3D avatars with words. ACM Trans Graph. 2016;35(4):1–14.

[46] Thaler A, Geuss MN, Mölbert SC, Giel KE, Streuber S, Romero J, et al. Body size estimation of self and others in females varying in BMI. PLoS ONE. 2018;13(2): e0192152. 29425218 10.1371/journal.pone.0192152 PMC5806871

[47] Vocks S, Legenbauer T, Wachter A, Wucherer M, Kosfelder J. What happens in the course of body exposure? Emotional, cognitive, and physiological reactions to mirror confrontation in eating disorders. J Psychosom Res. 2007;62(2):231–9. 17270582 10.1016/j.jpsychores.2006.08.007

[48] Voges MM, Giabbiconi CM, Schone B, Braks K, Huber TJ, Waldorf M, et al. Double standards in body evaluation? How identifying with a body stimulus influences ratings in women with anorexia nervosa and bulimia nervosa. Int J Eat Disord. 2018;51(11):1223–32. 30480829 10.1002/eat.22967

[49] von Collani G, Herzberg PY. Eine revidierte Fassung der deutschsprachigen Skala zum Selbstwertgefühl von Rosenberg. Zeitschrift Für Differentielle und Diagnostische Psychologie. 2003;24(1):3–7.

[50] Weinberger NA, Kersting A, Riedel-Heller SG, Luck-Sikorski C. Body dissatisfaction in individuals with obesity compared to normal-weight individuals: a systematic review and meta-analysis. Obes Facts. 2016;9(6):424–41. 28013298 10.1159/000454837 PMC5644896

