# Supplementary material for: Body image differs in weight-based stereotypes between patients with bulimia nervosa and binge eating disorder: findings from the BodyTalk project
Source: J Eat Disord. 2025 Feb 17;13:32. doi: 10.1186/s40337-025-01201-5 (PMC11834605; doi:10.1186/s40337-025-01201-5)
Supplement: Supplementary file 1 — Supplementary Material [file 40337_2025_1201_MOESM1_ESM.docx]

**Supplementary Material**

**Body image differs in weight-based stereotypes between patients with bulimia nervosa and binge eating disorder: Findings from the BodyTalk project**

**Schweda et al., 2024**

**Supplementary results: Primary analyses with BN- and BED-specific control groups**

In our main manuscript we report a set of analyses in which we compare linguistic body image representations between patients with bulimia nervosa, binge eating disorder, and an aggregated control group which consist of participants with normal weight, as well as participants who were overweight or obese (i.e. had a BMI > 25). This was done in order for the control group to be heterogeneous enough so that the effects produced cannot solely be attributable to the BMI of the patient groups. Yet, the groups were aggregated because the main focus of our hypotheses was placed on the eating disorder. To illuminate potential effects of the BMI, and to ensure that the results remain stable in a more granular analysis, a reproduction of the analyses reported in the manuscript, yet, differentiating the control participants suffering (overweight, OW) and not suffering from obesity (normal weight, NW) is reported in these supplemental documents. In supplemental table 1, we report and compare the respective group characteristics.

**Rating task**

In the main manuscript, we present results from a generalized estimating equations (GEE) model in which we regressed the valence of the respective adjective, the BMI of the 3D-avatar, and the group membership of the participant on the rating in the respective trial. Subsequently, we conducted marginal effects analyses to assess the differences between the group-specific linear slopes in the rating scores across BMI for each specific valence, as well as the predicted means at each BMI. We found that steeper curves for patients with BN at attribute valences of -2, 1 and 2, and a large set of differences in the predicted means that mainly occurring at higher BMIs at valences -2, 1 and 2 (see supplemental tables 2c and 2d). Here, we reproduced this analysis but classified participants into the above mentioned four instead of three groups. In the GEE, we again find a significant valence x BMI x group interaction (χ^2^(3) = 36, p < .001). Subsequent slope analyses reveal a similar pattern as in the main analyses (see supplemental figure 1). At valence 2, we find significant differences in the steepness of the slopes between the BN and the BED, the BN and the NW control group, as well as the BN and the OW control group. Here, the difference between the NW and OW control group also reaches significance. In the same fashion, the analyses reveal differences between the BN and the BED, the BN and the NW control group, as well as the BN and the OW control group. At a valence of -2, the BN group differs significantly from the BED group, the BN group from the OW control group and the NW control group from the OW control group. Yet, the latter pattern only occurs at an uncorrected alpha-level. Tukey-corrected marginal effects of predicted means at each level of the BMI reveal significant differences between the BN and the three other groups at valence = 2 largely for BMIs ≥ 18.3. At valence = 1, such differences occur at BMIs ≥ 22.3. Differences between BN and the remaining groups at valence -2 start occurring at BMIs of 20. Hence, and despite some minor differences which possibly come about due to statistical power and the number of statistical comparisons, a very similar pattern occurs when comparing all four groups in a non-aggregated fashion.

|  | BN | BED | Controls NW | Controls OW | p | Effect size (ƞ²) | Contrasts sig. |
| --- | --- | --- | --- | --- | --- | --- | --- |
|  |  |  |  |  |  |  |  |
| n | 22 | 22 | 22 | 22 |  |  |  |
|  |  |  |  |  |  |  |  |
| Age (M / SD) | 26.7 (9.71) | 34.7 (13.2) | 26.8 (9.28) | 37.8 (15.3) | .004 | .15 |  |
| BMI (M / SD) | 21.20 (2.67) | 37.86 (8.63) | 21.39 (2.57) | 37.62 (11.19) | <.001 | .57 | 1,3,4,6 |
| Rosenberg SES (M / SD) | 15.59 (4.70) | 14.57 (5.51) | 21.55 (5.33) | 21.56 (4.86) | <.001 | .30 | 2,3,4,5 |
| EDE-Q - Restraint (M / SD) | 2.59 (1.64) | 2.48 (1.71) | 0.62 (0.74) | 2.02 (1.23) | <.001 | .25 | 2,4,6 |
| EDE-Q - Eating Concern (M / SD) | 3.16 (1.54) | 2.57 (1.73) | 0.25 (0.33) | 0.44 (0.75) | <.001 | .53 | 2,3,4,5 |
| EDE-Q - Weight Concern (M / SD) | 3.25 (1.98) | 4.00 (1.02) | 0.84 (0.60) | 2.25 (1.40) | <.001 | .45 | 2,4,5,6 |
| EDE-Q - Shape Concern (M / SD) | 3.94 (1.75) | 4.74 (1.16) | 1.01 (0.78) | 2.72 (1.64) | <.001 | .52 | 2,3,4,5,6 |
| EDE-Q - Total Score (M / SD) | 3.24 (1.52) | 3.45 (1.14) | 0.68 (0.51) | 1.86 (1.03) | <.001 | .52 | 2,3,4,5,6 |
| PHQ-8 - Depression Score (M / SD) | 12.82 (5.76) | 14.41 (5.55) | 4.41 (2.42) | 4.73 (2.78) | <.001 | .53 | 2,3,4,5 |
| FPS - Fat Phobia (M / SD) | 49.23 (6.58) | 49.14 (7.59) | 49.91 (4.95) | 48.19 (5.52) | <.843 | .01 |  |
| FKB-20 – Perception of Body Dynamics | 29.23 (5.78) | 24.79 (7.33) | 36.77 (5.42) | 33.33 (7.53) | <.001 | .33 | 2,4,5 |
| FKB-20 – Negative Body Image | 35.32 (8.58)ᶜ | 39.27 (6.51) | 21.27 (7.51) | 31.24 (10.59) | <.001 | .40 | 2,4,5,6 |
|  |  |  |  |  |  |  |  |
|  |  |  |  |  |  |  |  |
| PACS - Physical appearance comparison (M / SD) | 17.41 (3.90) | 18.95 (2.94) | 15.41 (3.02) | 14.86 (3.36) | <.001 | .20 | 4,5 |
|  |  |  |  |  |  |  |  |

*Supplemental table 1: Participant characteristics.* EDE-Q: Eating Disorder Examination Questionnaire (Hilbert, et al., 2007); PHQ-9: Patient Health Questionnaire (Kroenke & Spitzer, 2002), FPS: Fat Phobia Scale (Bacon, et al., 2001), FKB-20: Body Image Questionnaire (Clement & Löwe, 1996), R-SES: Rosenberg Self-Esteem Scale (von Collani & Herzberg, 2003); EDI-2: Eating Disorder Inventory 2 (Garner, et al., 1983), PACS: Physical Appearance Comparison Scale (Mölbert, Hautzinger, et al., 2017) The first four columns show proportions, or means and standard deviations of the respective measure. The fifth column contains p-values of omnibus ANOVAs for comparisons between groups, and the sixth column contains the effect size (h²) from such analyses. In the seventh and final column we report results from a Tukey-corrected post hoc analysis. Here, the digits code the respective contrast with significant results (1 = BN vs. BED, 2 = BN vs. Control NW, 3 = BN vs Control OW, 4 = BED vs. Control NW, 5 = BED = vs. Control OW, 6 = Control NW vs. Control OW).

**
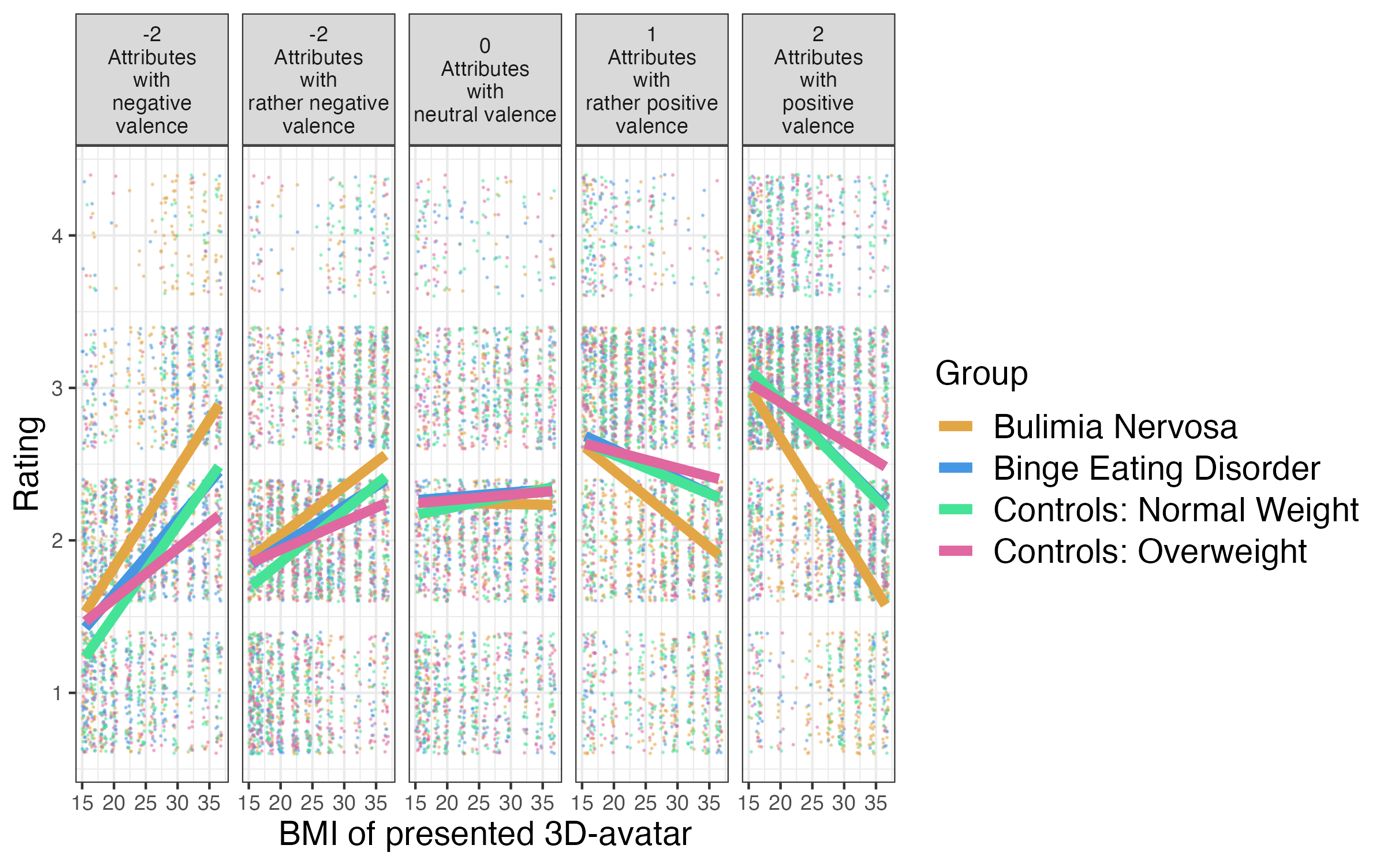
**

*Supplemental Figure 1.* Results of the rating task with four groups. Participants rated whether an attribute of a specific valence (e.g. friendly → positive valence) and an avatar with a specific body shape – operationalized via the BMI – fit together. This was done using a 4-point Likert-scale with the following levels: 1 = “not at all”; 2 = “rather not”, 3 = “rather yes”, 4 = “very much”. Despite some minor differences, the overall pattern remains the same as in the main manuscript.

**Body size estimation**

In the main manuscript, we reported group-wise differences in body size estimation as operationalized using the BPI. When both, the NW and the OW groups aggregated into one control group, we find that patients with BN overestimate their body size compared to the BED and the control group. Yet, and as mentioned in the manuscript, such differences are rather attributable to body weight. When including the unaggregated group classification with the NW and OW control groups, we find that the BN differs from the BED (overall: F(3, 83) = 7.14, η^2^ = .205, p < .001; specific contrast: p = .015), and the OW control group (p = .001), yet, no difference occurs between the BN and the NW control group (p = .870). Also, we find a significant difference between the BN and the BED group (p = .009). The results are illustrated in supplemental figure 2. Hence, BPIs are rather conditional on the participants’ weight, not the presence of BN and BED, which is in accordance with prior literature.


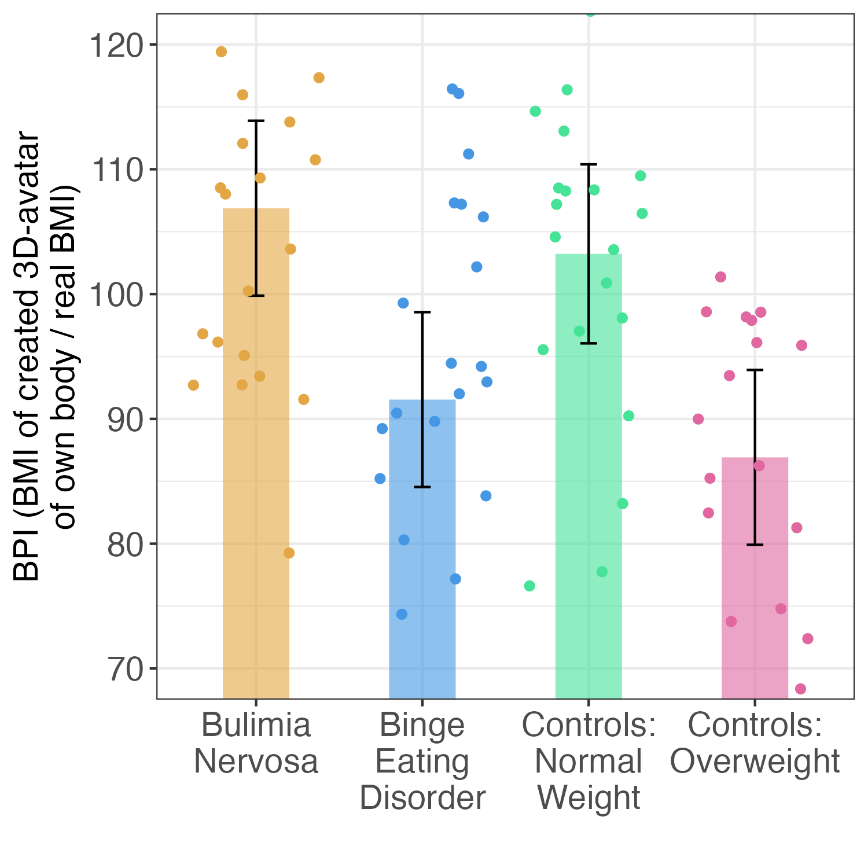


*Supplemental Figure 2.* Means, 95%-confidence intervals and raw data points of BPI values. Significant differences exist between the BN and the BED group, the NW and the OW controls, as well as the BN group and the OW controls.

**Adjustment task**

To analyze data from the adjustment task, we reported another GEE model in which we regressed the aggregated group variable and the valence of the specific attribute presented – including their interaction term – on the BMI of the 3D-avatar created by the participants. We reran these analyses with the disaggregated group variable (including the NW and OW control groups), again observing a significant interaction effect between valence and group (χ^2^ (2) = 15, p < .001, see supplemental figure 3). Tukey-corrected marginal slope analyses only reveal significant differences between the BN and the OW control group (p < .001). The comparisons between BN and BED, and BN and the NW control group approach the critical alpha level at p = .079 and p = .107, respectively. If these tests are not corrected for alpha-level inflation, both these comparisons yield significant results (p = .017 and p = .024). Also, there is a significant difference between the BED and the OW control group. On contrary, in the marginal effect analyses of the predicted means on each level of valence, significant differences between BN and all other groups occur at valences 1 and 2. Thus, and although we found some minor deviations from the analyses reported in the manuscript, which are again most likely due to power and the number of comparisons to be corrected for, we believe that the analyses reveal very similar patterns in terms of differential weight and valence representations in patients with bulimia nervosa.


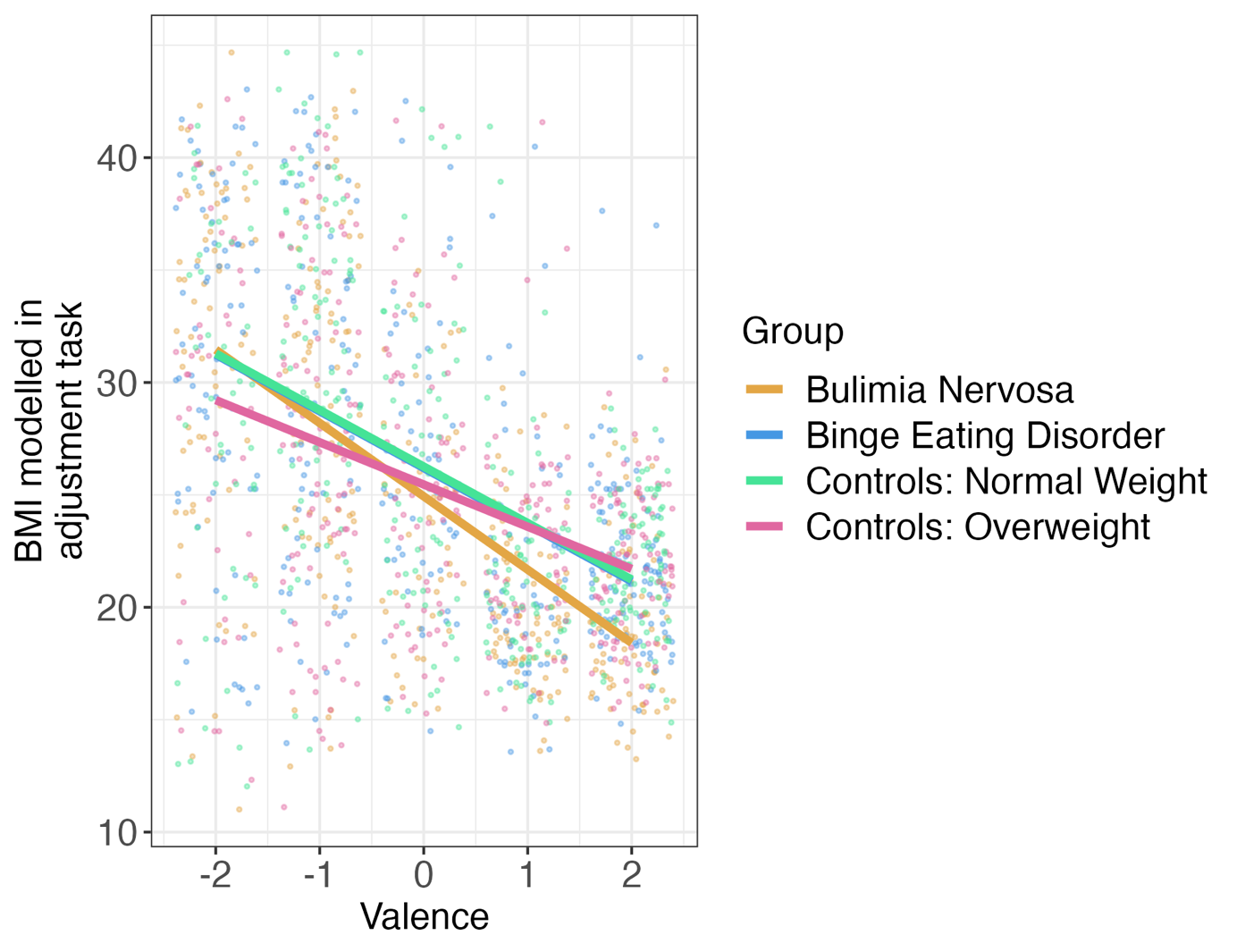


*Supplemental Figure 3.* Results from the GEE in the valence task. Participants created and shaped 3D-avatars based on attributes with different valences (e.g. friendly → positive valence). For the rating of the valences, we used a 5-point Likert-scale with the following descriptions: -2 = very negative; -1 = rather negative; 0 = neutral; 1 = rather positive; 2 = very positive. In marginal slope analyses, the slope of the BN group differs significantly from the other groups at an uncorrected alpha level. In Tukey-corrected comparisons between predicted means, the difference between the BN group becomes significant at valences 1 and 2.

**Supplemental results: Marginal effects of main analyses**

In our main manuscript we refrained from reporting each marginal effect for the sake of economic and comprehensible illustration of our results and readability. Here, we provide the interested reader with further results on each result. Tables 2a, 2b, 2c and 2d show the regression table, the results of the global variable-wise significance tests, the marginal slope and predicted mean analyses from the rating task. Tables 3a, 3b, 3c and 3d report the same outcomes for the GEE performed to analyze the adjustment task data.

|  | Estimate | Std.err | Wald | Pr(>\|W\|) |
| --- | --- | --- | --- | --- |
| (Intercept) | 2.26 | 0.0763 | 880 | < .001 |
| Valence | 0.867 | 0.0479 | 329 | < .001 |
| BMI | -0.000823 | 0.00311 | 0.0703 | 0.791 |
| Group: BED (ref. BN) | -0.0499 | 0.117 | 0.181 | 0.671 |
| Group: Control (ref. BN) | -0.148 | 0.101 | 2.17 | 0.141 |
| Valence x BMI | -0.0328 | 0.00204 | 258 | < .001 |
| Valence x Group: BED | -0.0964 | 0.0726 | 1.77 | 0.184 |
| Valence x Group: Controls | -0.122 | 0.0624 | 3.81 | 0.051 |
| BMI x Group: BED | 0.00422 | 0.00459 | 0.846 | 0.358 |
| BMI x Group: Controls | 0.00684 | 0.00399 | 2.95 | 0.086 |
| Valence x BMI x Group: BED | 0.0101 | 0.00296 | 11.5 | < .001 |
| Valence x BMI x Group: Controls | 0.0124 | 0.00258 | 22.9 | < .001 |

*Supplemental Table 2a.* Regression table containing results from the rating task GEE. Effects are treatment-coded.

|  | Df | X2 | P(>\|Chi\|) |
| --- | --- | --- | --- |
| Valence | 1 | 436 | 0 |
| BMI | 1 | 0.000599 | 0.98 |
| Group | 2 | 4.06 | 0.131 |
| Valence x BMI | 1 | 482 | 0 |
| Valence x Group | 2 | 90 | 0 |
| BMI x Group | 2 | 4.31 | 0.116 |
| Valence x BMI x Group | 2 | 23.8 | < .001 |

*Supplemental Table 2b*. Global variable-wise effects from the rating task GEE.

| contrast | Valence | estimate | SE | z | p-Value |
| --- | --- | --- | --- | --- | --- |
| BN - BED | -2 | 0.0159 | 0.00806 | 1.97 | 0.119 |
| BN - Controls | -2 | 0.0179 | 0.00714 | 2.51 | 0.0327 |
| BED - Controls | -2 | 0.002 | 0.00739 | 0.271 | 0.96 |
| BN - BED | -1 | 0.00584 | 0.00585 | 0.998 | 0.578 |
| BN - Controls | -1 | 0.00553 | 0.00518 | 1.07 | 0.534 |
| BED - Controls | -1 | -0.000312 | 0.00539 | -0.0579 | 0.998 |
| BN - BED | 0 | -0.00422 | 0.00459 | -0.92 | 0.628 |
| BN - Controls | 0 | -0.00684 | 0.00399 | -1.72 | 0.199 |
| BED - Controls | 0 | -0.00262 | 0.0042 | -0.625 | 0.806 |
| BN - BED | 1 | -0.0143 | 0.00504 | -2.84 | 0.0127 |
| BN - Controls | 1 | -0.0192 | 0.00428 | -4.49 | < .001 |
| BED - Controls | 1 | -0.00494 | 0.00453 | -1.09 | 0.52 |
| BN - BED | 2 | -0.0243 | 0.00687 | -3.54 | < .001 |
| BN - Controls | 2 | -0.0316 | 0.00584 | -5.41 | < .001 |
| BED - Controls | 2 | -0.00725 | 0.00614 | -1.18 | 0.464 |

*Supplemental Table 2c.* Tukey-corrected marginal slope comparisons for the rating task. Correction is always performed for families of three estimates.

| contrast | Valence | BMI | estimate | SE | z | p-Value |
| --- | --- | --- | --- | --- | --- | --- |
| BN - BED | -2 | 15.5 | 0.103 | 0.0896 | 1.16 | 0.48 |
| BN - Controls | -2 | 15.5 | 0.182 | 0.077 | 2.36 | 0.0476 |
| BED - Controls | -2 | 15.5 | 0.0784 | 0.0864 | 0.908 | 0.635 |
| BN - BED | -1 | 15.5 | 0.044 | 0.0665 | 0.662 | 0.786 |
| BN - Controls | -1 | 15.5 | 0.112 | 0.0568 | 1.97 | 0.12 |
| BED - Controls | -1 | 15.5 | 0.0679 | 0.0643 | 1.06 | 0.541 |
| BN - BED | 0 | 15.5 | -0.0154 | 0.053 | -0.291 | 0.954 |
| BN - Controls | 0 | 15.5 | 0.0419 | 0.045 | 0.932 | 0.62 |
| BED - Controls | 0 | 15.5 | 0.0574 | 0.0505 | 1.14 | 0.492 |
| BN - BED | 1 | 15.5 | -0.0749 | 0.0564 | -1.33 | 0.379 |
| BN - Controls | 1 | 15.5 | -0.0281 | 0.0482 | -0.582 | 0.83 |
| BED - Controls | 1 | 15.5 | 0.0469 | 0.0522 | 0.898 | 0.642 |
| BN - BED | 2 | 15.5 | -0.134 | 0.0743 | -1.81 | 0.167 |
| BN - Controls | 2 | 15.5 | -0.098 | 0.0642 | -1.53 | 0.278 |
| BED - Controls | 2 | 15.5 | 0.0363 | 0.0681 | 0.533 | 0.855 |
| BN - BED | -2 | 16.7 | 0.122 | 0.0823 | 1.48 | 0.298 |
| BN - Controls | -2 | 16.7 | 0.203 | 0.0709 | 2.86 | 0.0117 |
| BED - Controls | -2 | 16.7 | 0.0808 | 0.0791 | 1.02 | 0.564 |
| BN - BED | -1 | 16.7 | 0.0509 | 0.0612 | 0.832 | 0.683 |
| BN - Controls | -1 | 16.7 | 0.118 | 0.0523 | 2.26 | 0.0608 |
| BED - Controls | -1 | 16.7 | 0.0675 | 0.059 | 1.15 | 0.486 |
| BN - BED | 0 | 16.7 | -0.0204 | 0.0489 | -0.418 | 0.908 |
| BN - Controls | 0 | 16.7 | 0.0339 | 0.0415 | 0.816 | 0.693 |
| BED - Controls | 0 | 16.7 | 0.0543 | 0.0465 | 1.17 | 0.473 |
| BN - BED | 1 | 16.7 | -0.0918 | 0.052 | -1.76 | 0.182 |
| BN - Controls | 1 | 16.7 | -0.0507 | 0.0447 | -1.14 | 0.492 |
| BED - Controls | 1 | 16.7 | 0.041 | 0.0481 | 0.854 | 0.669 |
| BN - BED | 2 | 16.7 | -0.163 | 0.0686 | -2.38 | 0.0458 |
| BN - Controls | 2 | 16.7 | -0.135 | 0.0596 | -2.27 | 0.0599 |
| BED - Controls | 2 | 16.7 | 0.0278 | 0.0626 | 0.444 | 0.897 |
| BN - BED | -2 | 18.3 | 0.148 | 0.0731 | 2.03 | 0.106 |
| BN - Controls | -2 | 18.3 | 0.232 | 0.0634 | 3.66 | <.001 |
| BED - Controls | -2 | 18.3 | 0.0841 | 0.0696 | 1.21 | 0.449 |
| BN - BED | -1 | 18.3 | 0.0605 | 0.0544 | 1.11 | 0.507 |
| BN - Controls | -1 | 18.3 | 0.128 | 0.0466 | 2.73 | 0.0172 |
| BED - Controls | -1 | 18.3 | 0.067 | 0.052 | 1.29 | 0.402 |
| BN - BED | 0 | 18.3 | -0.0273 | 0.0436 | -0.627 | 0.805 |
| BN - Controls | 0 | 18.3 | 0.0226 | 0.0371 | 0.61 | 0.814 |
| BED - Controls | 0 | 18.3 | 0.05 | 0.0412 | 1.21 | 0.445 |
| BN - BED | 1 | 18.3 | -0.115 | 0.0466 | -2.47 | 0.0361 |
| BN - Controls | 1 | 18.3 | -0.0822 | 0.0402 | -2.04 | 0.102 |
| BED - Controls | 1 | 18.3 | 0.0329 | 0.0428 | 0.77 | 0.721 |
| BN - BED | 2 | 18.3 | -0.203 | 0.0615 | -3.3 | 0.003 |
| BN - Controls | 2 | 18.3 | -0.187 | 0.0539 | -3.47 | 0.002 |
| BED - Controls | 2 | 18.3 | 0.0159 | 0.0557 | 0.286 | 0.956 |
| BN - BED | -2 | 20 | 0.175 | 0.0653 | 2.67 | 0.0206 |
| BN - Controls | -2 | 20 | 0.262 | 0.0573 | 4.57 | <.001 |
| BED - Controls | -2 | 20 | 0.0874 | 0.061 | 1.43 | 0.325 |
| BN - BED | -1 | 20 | 0.0701 | 0.0485 | 1.44 | 0.318 |
| BN - Controls | -1 | 20 | 0.137 | 0.0419 | 3.26 | 0.003 |
| BED - Controls | -1 | 20 | 0.0665 | 0.0457 | 1.46 | 0.312 |
| BN - BED | 0 | 20 | -0.0343 | 0.0391 | -0.878 | 0.654 |
| BN - Controls | 0 | 20 | 0.0113 | 0.0333 | 0.34 | 0.938 |
| BED - Controls | 0 | 20 | 0.0457 | 0.0364 | 1.25 | 0.422 |
| BN - BED | 1 | 20 | -0.139 | 0.0422 | -3.29 | 0.003 |
| BN - Controls | 1 | 20 | -0.114 | 0.0366 | -3.11 | .005 |
| BED - Controls | 1 | 20 | 0.0248 | 0.0382 | 0.65 | 0.793 |
| BN - BED | 2 | 20 | -0.243 | 0.0558 | -4.36 | <.001 |
| BN - Controls | 2 | 20 | -0.239 | 0.0495 | -4.83 | <.001 |
| BED - Controls | 2 | 20 | 0.00395 | 0.0497 | 0.0794 | 0.997 |
| BN - BED | -2 | 22.3 | 0.212 | 0.0578 | 3.67 | <.001 |
| BN - Controls | -2 | 22.3 | 0.304 | 0.0521 | 5.83 | <.001 |
| BED - Controls | -2 | 22.3 | 0.092 | 0.0514 | 1.79 | 0.173 |
| BN - BED | -1 | 22.3 | 0.0838 | 0.0426 | 1.97 | 0.121 |
| BN - Controls | -1 | 22.3 | 0.15 | 0.0377 | 3.97 | <.001 |
| BED - Controls | -1 | 22.3 | 0.0658 | 0.0384 | 1.71 | 0.2 |
| BN - BED | 0 | 22.3 | -0.0442 | 0.0345 | -1.28 | 0.406 |
| BN - Controls | 0 | 22.3 | -0.00466 | 0.0298 | -0.157 | 0.987 |
| BED - Controls | 0 | 22.3 | 0.0395 | 0.0311 | 1.27 | 0.412 |
| BN - BED | 1 | 22.3 | -0.172 | 0.0381 | -4.52 | <.001 |
| BN - Controls | 1 | 22.3 | -0.159 | 0.0335 | -4.75 | <.001 |
| BED - Controls | 1 | 22.3 | 0.0132 | 0.0335 | 0.396 | 0.917 |
| BN - BED | 2 | 22.3 | -0.3 | 0.051 | -5.89 | <.001 |
| BN - Controls | 2 | 22.3 | -0.313 | 0.046 | -6.81 | <.001 |
| BED - Controls | 2 | 22.3 | -0.013 | 0.044 | -0.296 | 0.953 |
| BN - BED | -2 | 24.3 | 0.243 | 0.0558 | 4.37 | <.001 |
| BN - Controls | -2 | 24.3 | 0.339 | 0.0517 | 6.57 | <.001 |
| BED - Controls | -2 | 24.3 | 0.096 | 0.0468 | 2.05 | 0.1 |
| BN - BED | -1 | 24.3 | 0.0954 | 0.0407 | 2.34 | 0.0499 |
| BN - Controls | -1 | 24.3 | 0.161 | 0.0369 | 4.36 | <.001 |
| BED - Controls | -1 | 24.3 | 0.0652 | 0.0347 | 1.88 | 0.146 |
| BN - BED | 0 | 24.3 | -0.0526 | 0.0329 | -1.6 | 0.247 |
| BN - Controls | 0 | 24.3 | -0.0183 | 0.0288 | -0.634 | 0.801 |
| BED - Controls | 0 | 24.3 | 0.0343 | 0.0285 | 1.2 | 0.452 |
| BN - BED | 1 | 24.3 | -0.201 | 0.0372 | -5.38 | <.001 |
| BN - Controls | 1 | 24.3 | -0.197 | 0.033 | -5.98 | <.001 |
| BED - Controls | 1 | 24.3 | 0.00343 | 0.0318 | 0.108 | 0.994 |
| BN - BED | 2 | 24.3 | -0.349 | 0.0507 | -6.88 | <.001 |
| BN - Controls | 2 | 24.3 | -0.376 | 0.0461 | -8.15 | <.001 |
| BED - Controls | 2 | 24.3 | -0.0274 | 0.0425 | -0.646 | 0.795 |
| BN - BED | -2 | 26.1 | 0.271 | 0.0577 | 4.7 | <.001 |
| BN - Controls | -2 | 26.1 | 0.371 | 0.0545 | 6.81 | <.001 |
| BED - Controls | -2 | 26.1 | 0.0995 | 0.0464 | 2.15 | 0.0809 |
| BN - BED | -1 | 26.1 | 0.106 | 0.0417 | 2.53 | 0.0303 |
| BN - Controls | -1 | 26.1 | 0.17 | 0.0385 | 4.42 | <.001 |
| BED - Controls | -1 | 26.1 | 0.0646 | 0.0341 | 1.9 | 0.14 |
| BN - BED | 0 | 26.1 | -0.06 | 0.0336 | -1.79 | 0.174 |
| BN - Controls | 0 | 26.1 | -0.0303 | 0.0298 | -1.02 | 0.566 |
| BED - Controls | 0 | 26.1 | 0.0297 | 0.0282 | 1.05 | 0.543 |
| BN - BED | 1 | 26.1 | -0.226 | 0.0387 | -5.83 | <.001 |
| BN - Controls | 1 | 26.1 | -0.231 | 0.0344 | -6.72 | <.001 |
| BED - Controls | 1 | 26.1 | -0.00526 | 0.0324 | -0.162 | 0.986 |
| BN - BED | 2 | 26.1 | -0.391 | 0.0534 | -7.32 | <.001 |
| BN - Controls | 2 | 26.1 | -0.432 | 0.0486 | -8.88 | <.001 |
| BED - Controls | 2 | 26.1 | -0.0402 | 0.044 | -0.914 | 0.631 |
| BN - BED | -2 | 27.8 | 0.299 | 0.0629 | 4.76 | <.001 |
| BN - Controls | -2 | 27.8 | 0.402 | 0.0598 | 6.73 | <.001 |
| BED - Controls | -2 | 27.8 | 0.103 | 0.0495 | 2.08 | 0.0936 |
| BN - BED | -1 | 27.8 | 0.116 | 0.0451 | 2.57 | 0.0274 |
| BN - Controls | -1 | 27.8 | 0.18 | 0.0421 | 4.28 | <.001 |
| BED - Controls | -1 | 27.8 | 0.0641 | 0.036 | 1.78 | 0.176 |
| BN - BED | 0 | 27.8 | -0.0674 | 0.0361 | -1.87 | 0.149 |
| BN - Controls | 0 | 27.8 | -0.0423 | 0.0323 | -1.31 | 0.389 |
| BED - Controls | 0 | 27.8 | 0.0251 | 0.0297 | 0.844 | 0.675 |
| BN - BED | 1 | 27.8 | -0.251 | 0.0421 | -5.96 | <.001 |
| BN - Controls | 1 | 27.8 | -0.265 | 0.0372 | -7.11 | <.001 |
| BED - Controls | 1 | 27.8 | -0.0139 | 0.0349 | -0.398 | 0.916 |
| BN - BED | 2 | 27.8 | -0.434 | 0.0586 | -7.41 | <.001 |
| BN - Controls | 2 | 27.8 | -0.487 | 0.053 | -9.19 | <.001 |
| BED - Controls | 2 | 27.8 | -0.0529 | 0.0479 | -1.1 | 0.512 |
| BN - BED | -2 | 29.6 | 0.327 | 0.0705 | 4.63 | <.001 |
| BN - Controls | -2 | 29.6 | 0.433 | 0.067 | 6.47 | <.001 |
| BED - Controls | -2 | 29.6 | 0.107 | 0.0555 | 1.92 | 0.133 |
| BN - BED | -1 | 29.6 | 0.126 | 0.0503 | 2.51 | 0.0328 |
| BN - Controls | -1 | 29.6 | 0.19 | 0.0471 | 4.02 | <.001 |
| BED - Controls | -1 | 29.6 | 0.0635 | 0.04 | 1.59 | 0.251 |
| BN - BED | 0 | 29.6 | -0.0747 | 0.0401 | -1.87 | 0.149 |
| BN - Controls | 0 | 29.6 | -0.0542 | 0.0359 | -1.51 | 0.287 |
| BED - Controls | 0 | 29.6 | 0.0205 | 0.0328 | 0.625 | 0.806 |
| BN - BED | 1 | 29.6 | -0.276 | 0.0468 | -5.89 | <.001 |
| BN - Controls | 1 | 29.6 | -0.298 | 0.0412 | -7.23 | <.001 |
| BED - Controls | 1 | 29.6 | -0.0225 | 0.0388 | -0.58 | 0.831 |
| BN - BED | 2 | 29.6 | -0.476 | 0.0655 | -7.27 | <.001 |
| BN - Controls | 2 | 29.6 | -0.542 | 0.0587 | -9.22 | <.001 |
| BED - Controls | 2 | 29.6 | -0.0655 | 0.0537 | -1.22 | 0.442 |
| BN - BED | -2 | 32.1 | 0.368 | 0.0849 | 4.33 | <.001 |
| BN - Controls | -2 | 32.1 | 0.48 | 0.0802 | 5.98 | <.001 |
| BED - Controls | -2 | 32.1 | 0.112 | 0.068 | 1.64 | 0.228 |
| BN - BED | -1 | 32.1 | 0.141 | 0.0605 | 2.33 | 0.0512 |
| BN - Controls | -1 | 32.1 | 0.204 | 0.0565 | 3.61 | <.001 |
| BED - Controls | -1 | 32.1 | 0.0627 | 0.0488 | 1.28 | 0.404 |
| BN - BED | 0 | 32.1 | -0.0856 | 0.0478 | -1.79 | 0.173 |
| BN - Controls | 0 | 32.1 | -0.0719 | 0.0429 | -1.68 | 0.214 |
| BED - Controls | 0 | 32.1 | 0.0137 | 0.0396 | 0.347 | 0.936 |
| BN - BED | 1 | 32.1 | -0.312 | 0.0557 | -5.61 | <.001 |
| BN - Controls | 1 | 32.1 | -0.348 | 0.0487 | -7.14 | <.001 |
| BED - Controls | 1 | 32.1 | -0.0353 | 0.0465 | -0.758 | 0.729 |
| BN - BED | 2 | 32.1 | -0.539 | 0.0781 | -6.91 | <.001 |
| BN - Controls | 2 | 32.1 | -0.624 | 0.0693 | -9 | <.001 |
| BED - Controls | 2 | 32.1 | -0.0843 | 0.0647 | -1.3 | 0.393 |
| BN - BED | -2 | 34.4 | 0.404 | 0.0994 | 4.06 | <.001 |
| BN - Controls | -2 | 34.4 | 0.52 | 0.0931 | 5.59 | <.001 |
| BED - Controls | -2 | 34.4 | 0.116 | 0.0811 | 1.43 | 0.324 |
| BN - BED | -1 | 34.4 | 0.154 | 0.0708 | 2.18 | 0.0747 |
| BN - Controls | -1 | 34.4 | 0.216 | 0.0658 | 3.29 | 0.003 |
| BED - Controls | -1 | 34.4 | 0.062 | 0.0582 | 1.07 | 0.536 |
| BN - BED | 0 | 34.4 | -0.0952 | 0.0558 | -1.7 | 0.203 |
| BN - Controls | 0 | 34.4 | -0.0874 | 0.0499 | -1.75 | 0.187 |
| BED - Controls | 0 | 34.4 | 0.00779 | 0.0467 | 0.167 | 0.985 |
| BN - BED | 1 | 34.4 | -0.345 | 0.0646 | -5.34 | <.001 |
| BN - Controls | 1 | 34.4 | -0.391 | 0.0562 | -6.95 | <.001 |
| BED - Controls | 1 | 34.4 | -0.0464 | 0.0544 | -0.853 | 0.67 |
| BN - BED | 2 | 34.4 | -0.594 | 0.0905 | -6.57 | 1<.001 |
| BN - Controls | 2 | 34.4 | -0.695 | 0.0797 | -8.73 | <.001 |
| BED - Controls | 2 | 34.4 | -0.101 | 0.0757 | -1.33 | 0.379 |
| BN - BED | -2 | 36.5 | 0.437 | 0.114 | 3.84 | <.001 |
| BN - Controls | -2 | 36.5 | 0.557 | 0.106 | 5.27 | <.001 |
| BED - Controls | -2 | 36.5 | 0.12 | 0.0941 | 1.28 | 0.407 |
| BN - BED | -1 | 36.5 | 0.167 | 0.0811 | 2.05 | 0.0998 |
| BN - Controls | -1 | 36.5 | 0.228 | 0.075 | 3.04 | 0.007 |
| BED - Controls | -1 | 36.5 | 0.0614 | 0.0676 | 0.908 | 0.636 |
| BN - BED | 0 | 36.5 | -0.104 | 0.0638 | -1.63 | 0.233 |
| BN - Controls | 0 | 36.5 | -0.102 | 0.0569 | -1.79 | 0.174 |
| BED - Controls | 0 | 36.5 | 0.00233 | 0.054 | 0.0433 | 0.999 |
| BN - BED | 1 | 36.5 | -0.374 | 0.0734 | -5.1 | <.001 |
| BN - Controls | 1 | 36.5 | -0.431 | 0.0637 | -6.77 | <.001 |
| BED - Controls | 1 | 36.5 | -0.0567 | 0.0623 | -0.91 | 0.634 |
| BN - BED | 2 | 36.5 | -0.645 | 0.103 | -6.28 | <.001 |
| BN - Controls | 2 | 36.5 | -0.761 | 0.0899 | -8.46 | <.001 |
| BED - Controls | 2 | 36.5 | -0.116 | 0.0865 | -1.34 | 0.374 |

*Supplemental Table 2d.* Tukey-corrected predicted means comparisons for the rating task. Correction is always performed for families of three estimates.

|  | Estimate | Std.err | Wald | Pr(>\|W\|) |
| --- | --- | --- | --- | --- |
| (Intercept) | 24.9 | 0.316 | 6230 | <.001 |
| Valence | -3.26 | 0.22 | 221 | <.001 |
| Group: BED | 1.26 | 0.472 | 7.16 | .007 |
| Group: Controls | 0.917 | 0.406 | 5.09 | 0.024 |
| Valence x Group: BED | 0.741 | 0.31 | 5.72 | 0.0168 |
| Valence x Group: Controls | 1.07 | 0.278 | 14.7 | <.001 |

*Supplemental Table 3a.* Regression table containing results from the adjustment task GEE. Effects are treatment-coded.

|  | Df | X2 | P(>\|Chi\|) |
| --- | --- | --- | --- |
| Valence | 1 | 487 | < .001 |
| Group | 2 | 9.97 | 0.00685 |
| Valence x Group | 2 | 14.8 | 0.000609 |

*Supplemental Table 3b.* Global variable-wise effects from the adjustment task GEE.

| contrast | estimate | SE | z | p-Value |
| --- | --- | --- | --- | --- |
| BN - BED | -0.741 | 0.31 | -2.39 | 0.0442 |
| BN - Controls | -1.07 | 0.278 | -3.84 | < .001 |
| BED - Controls | -0.325 | 0.277 | -1.17 | 0.469 |

*Supplemental Table 3c.* Tukey-corrected marginal slope comparisons for the adjustment task. Correction is performed for families of three estimates.

| contrast | Valence | estimate | SE | z | p-Value |
| --- | --- | --- | --- | --- | --- |
| BN - BED | -2 | 0.22 | 0.963 | 0.228 | 0.972 |
| BN - Controls | -2 | 1.22 | 0.866 | 1.4 | 0.339 |
| BED - Controls | -2 | 0.996 | 0.88 | 1.13 | 0.495 |
| BN - BED | -1 | -0.521 | 0.692 | -0.753 | 0.732 |
| BN - Controls | -1 | 0.149 | 0.617 | 0.242 | 0.968 |
| BED - Controls | -1 | 0.671 | 0.636 | 1.05 | 0.543 |
| BN - BED | 0 | -1.26 | 0.472 | -2.68 | 0.0204 |
| BN - Controls | 0 | -0.917 | 0.406 | -2.26 | 0.0621 |
| BED - Controls | 0 | 0.346 | 0.434 | 0.797 | 0.705 |
| BN - BED | 1 | -2 | 0.398 | -5.04 | <.001 |
| BN - Controls | 1 | -1.98 | 0.323 | -6.14 | <.001 |
| BED - Controls | 1 | 0.0207 | 0.353 | 0.0586 | 0.998 |
| BN - BED | 2 | -2.74 | 0.535 | -5.13 | <.001 |
| BN - Controls | 2 | -3.05 | 0.445 | -6.85 | <.001 |
| BED - Controls | 2 | -0.304 | 0.464 | -0.656 | 0.789 |

*Supplemental Table 3d.* Tukey-corrected predicted means comparisons for the adjustment task. Correction is always performed for families of three estimates.

**Supplemental results: Manipulation check**

In the manuscript, we report that patients with bulimia nervosa tend rate the attributes (i.e. our stimuli) in an overall less positive fashion. Yet, such effects are small, and there is no systematic, stimulus-specific bias in this group. Also, we report that, using the attributes at hand, we succeeded at creating a heterogeneity of differently rated stimuli. Supplemental figure 4, hence, illustrates the group-wise and stimulus-wise differences in a priori valence ratings.


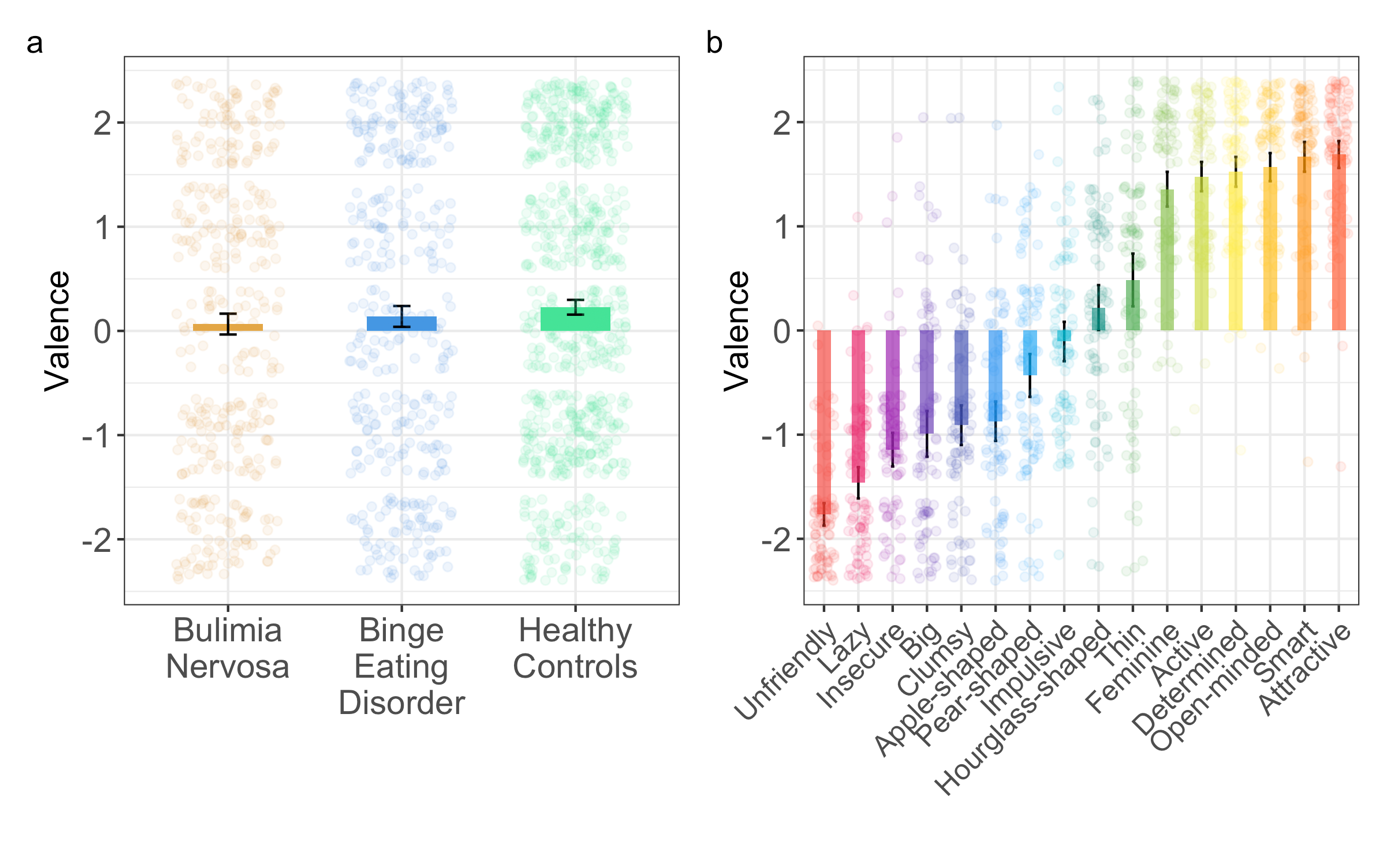


*Supplemental Figure 4.* A priori valence ratings of our stimuli. For the rating of the valences, we used a 5-point Likert-scale with the following descriptions: -2 = very negative; -1 = rather negative; 0 = neutral; 1 = rather positive; 2 = very positive. The left panel presents group-wise differences between the BN and the other two groups. The bars represent means with 95% confidence intervals. The dots show the raw data. The BN group exhibits overall less positive ratings of the attributes, yet, this effect is small (η² = .01), and there is no group x attribute interaction. The right panel shows the means, the 95% confidence interval and the raw data for the a priori valence ratings of each attribute. Indeed, it appears that we succeeded at creating a stimulus set that captures positive, neutral and negative valences equally well.

**Supplemental results: Comparison between German and Italian subsample**

To further explore potential differences between the German and Italian subsamples, we compared their sociodemographic characteristics and baseline variables, as shown in supplemental table 4. It became evident that the German subsample contained a larger proportion of participants from the BED group, although this difference was not significant in a Fisher’s exact test. A significant difference emerged, however, when comparing BMI between the two subsamples using a Mann-Whitney U-test. This difference was reduced when we conducted a two-way ANOVA including both subsample (German vs. Italian) and group (AN vs. BN vs. BED) as factors. The effect dropped from F(1, 86) = 9.07, p = .003 when considering subsample alone to F(1, 82) = 3.82, p = .054 when both factors were included. A similar outcome was observed using a robust two-way ANOVA from the WRS2 package (Mair & Wilcox, 2020). These results suggest that the BMI difference is largely driven by the higher proportion of BED patients in the German sample.

We also investigated whether the adjectives used as stimuli in the rating and adjustment tasks were evaluated differently in terms of their valence or elicited differences in the avatars generated. Before starting the main experimental tasks, participants rated each adjective for its valence. To measure the reliability of these ratings between samples, we calculated the average valence rating per adjective for each group and conducted a correlation analysis. The result, r = .966 (t(14) = 14, p < .001), indicates strong concordance, comparable to very good test-retest reliability (above .9). A similar analysis was conducted for the adjustment task, where we examined whether the average avatar size, operationalized by BMI, differed between samples. Again, we observed a strong correlation between the German and Italian samples (r = .913, t(8) = 14, p < .001).

To substantiate these analyses and further investigate the influence of recruitment location, we examined whether specific adjectives were rated differently in terms of their valence or associated differently in terms of the generated avatar’s voluminosity. For the initial valence ratings, we reran an ANOVA with valence as the dependent variable, group (BN vs. BED vs. Control) and recruitment site (Germany vs. Italy) as between-subject factors, and adjective as the within-subject factor. A significant interaction between country and adjective (F(6.66, 546.26) = 3.59, p < .001) indicated that some adjectives were rated differently across subsamples. Follow-up analyses identified differences for three adjectives: thin, smart, and big. To test whether this affected the overall findings, we reanalyzed the data excluding trials with these adjectives. The removal did not change the main result, as the significant three-way interaction between valence, group, and BMI remained (χ²(2) = 22, p < .001).

A similar approach was applied to the adjustment task. We reran the model reported in the manuscript, removing the group term (due to convergence issues) and including adjective as a fixed term. Again, a significant interaction between country and adjective was observed (χ²(15) = 57, p < .001), with three adjectives—attractive, heavyset, and clumsy—showing differences in avatar size between samples. Removing these adjectives from the analysis did not alter the main result (valence x group interaction: χ²(2) = 15.7, p = .00039). These findings indicate that the overall results are not driven by differences in ratings or associations with specific stimuli.

To assess whether the main findings depended on recruitment site, we reran the GEE models, including subsample (German vs. Italian) as a categorical predictor. For the rating task, the valence x BMI x group interaction remained significant (F(2) = 12, χ² = 24, p < .001), but a four-way interaction involving valence, BMI, group, and subsample also emerged (F(2) = 6, χ² = 11, p = .003). This suggests that the former three-way interaction differs slightly between recruitment sites. Simple slope analyses revealed somewhat different patterns: in the German subsample, results closely mirrored those in the manuscript, with steeper slopes for the BN group at higher valences. For valence 2, significant differences were observed between BN and BED (estimate = -0.0344, z = -4.25, p < .0001) and BN and Controls (estimate = -0.0241, z = -3.41, p = 0.002). A similar pattern emerged for valence 1. In the Italian subsample, steeper slopes were observed for both eating disorder groups compared to the control group at valence 2 and 1. These results are detailed in figure 5.

Comparing specific BMI levels further clarified these findings. In the German subsample, the most prominent differences occurred between BN and other groups at valences 1 and 2 for BMIs ≥ 22.3. For the Italian subsample, differences emerged between BED and BN, BED and Controls, and BN and Controls at higher BMI levels, particularly 36.5 for valence 2. At valence 1 and a BMI of 36.5, significant differences were observed only between BED and Controls. These supplementary analyses support the manuscript's conclusion that patients with BN exhibit the steepest negative relationship between valence and BMI, particularly at high valences. However, the results also hint at a similar, albeit weaker, pattern in BED patients, particularly in the Italian sample. This has been added to the discussion and supplemental online material.

For the adjustment task, the valence x group interaction remained stable (p = 0.0006), with no main effects or interactions involving subsample (all p > .17). For the body size estimation task, we observed a marginal difference between subsamples (p = .064), with Italian participants showing slightly higher values. Adding subsample as a factor did not alter the group differences.


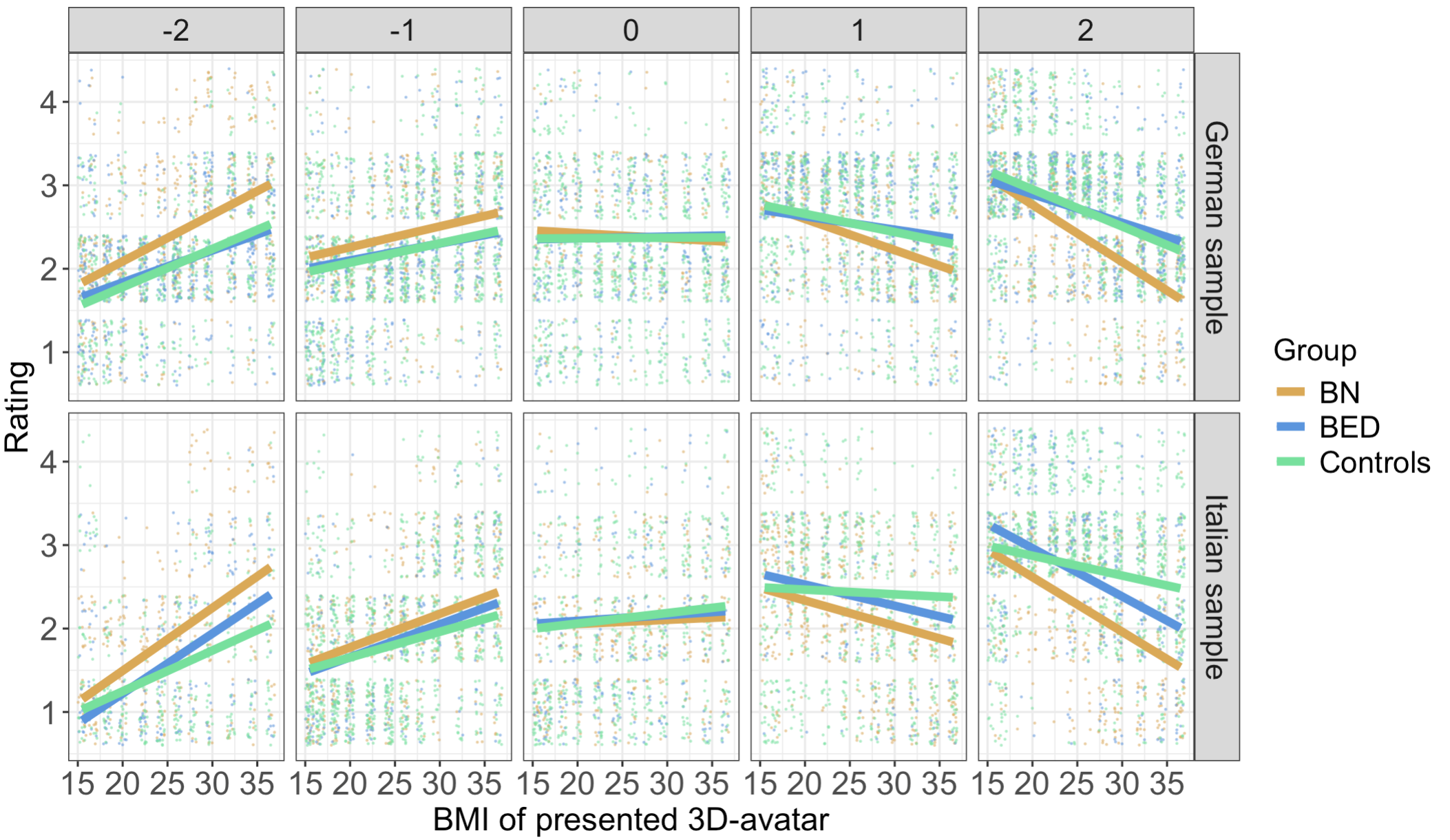


*Supplemental figure 5.* Main results from the rating task by subsample (German vs. Italian sample)

| Variable | Level | German Sample | |  | Italian Sample | | p |
| --- | --- | --- | --- | --- | --- | --- | --- |
| n |  | 50 | |  | 38 | |  |
|  |  |  |  |  |  |  |  |
| Sex | Female | 45 (90.0) | |  | 12 (100.0) | | 0,573 |
|  | Male | 5 (10.0) | |  | 0 (0.0) | |  |
| Group | BED | 15 (30.0) | |  | 7 (18.4) | | 0,43 |
|  | BN | 11 (22.0) | |  | 11 (28.9) | |  |
|  | Controls | 24 (48.0) | |  | 20 (52.6) | |  |
|  |  |  |  |  |  |  |  |
|  |  | Mean [SD) | Median [IQR] |  | Mean [SD) | Median [IQR] |  |
| Age |  | 33.2 (12.62) | 29.00 [23.00, 43.00] |  | 29.21 (13.04) | 24.00 [21.25, 31.50] | 0,052 |
| BMI |  | 32.45 (12.14) | 28.49 [20.85, 41.68] |  | 25.66 (7.74) | 24.40 [20.08, 29.40] | 0,019 |
| PHQ-D |  | 10.1 (7.29) | 8.00 [4.00, 16.75] |  | 7.76 (4.46) | 7.00 [4.00, 10.75] | 0,317 |
| PACS |  | 16.36 (3.73) | 17.00 [13.08, 19.00] |  | 17.05 (3.56) | 17.00 [14.25, 19.75] | 0,434 |
| FPS |  | 49.57 (7.53) | 49.00 [44.00, 54.00] |  | 48.55 (3.78) | 48.00 [46.25, 50.75] | 0,583 |
| EDE-Q - Weight Concern |  | 2.92 (1.81) | 3.35 [1.10, 4.40] |  | 2.15 (1.67) | 1.60 [0.80, 3.40] | 0,059 |
| EDE-Q - Shape Concern |  | 3.36 (2) | 3.81 [1.31, 5.09] |  | 2.77 (1.89) | 2.38 [1.28, 4.88] | 0,171 |
| EDE-Q - Eating Concern |  | 1.62 (1.81) | 0.60 [0.20, 2.60] |  | 1.59 (1.72) | 0.80 [0.20, 2.65] | 0,806 |
| EDE-Q - Restraint |  | 2.18 (1.62) | 2.00 [0.85, 3.55] |  | 1.59 (1.46) | 1.40 [0.40, 2.55] | 0,094 |
| EDE-Q - Total |  | 2.52 (1.62) | 2.63 [0.95, 3.65] |  | 2.03 (1.46) | 1.67 [0.88, 3.02] | 0,171 |
| FKB-20 - AKB |  | 33.27 (12.7) | 35.00 [21.00, 45.00] |  | 29.87 (7.02) | 30.00 [25.00, 33.00] | 0,092 |
| FKB-20 - VKD |  | 30.56 (8.56) | 31.11 [24.44, 36.00] |  | 31.58 (6.99) | 32.00 [28.25, 36.75] | 0,616 |
| GSES |  | 18.5 (7.63) | 19.00 [11.00, 25.75] |  | 18.08 (2.71) | 18.00 [16.00, 19.75] | 0.535 |

*Supplemental Table 4.* Comparison between the German and the Italian sample.

**Supplemental results: Approaching the design’s statistical power**

For the rating task, we conducted a simulation-based power analysis using Python. For that, we generated the variables that were being used as predictors using the experiment’s the design. Thus, all 12 avatars were shown to each participant in combination with all adjectives being pre-rated with valences of -2 to 2. The dependent variable *Rating* was generated using a simple regression function including all regression coefficients from the original models, except for the coefficient of interest, i.e. the three-way interaction between the BMI of the avatar, the valence of the adjective, and the group. Here, we chose an array of possible and realistic regression coefficients and simulated the probability of a significant three-way interaction 200 times for each combination, using GEE models with an exchangeable correlation structure. Variation was implemented by sampling from a normal distribution with the standard deviation of the residuals of the original model in each iteration, as well as sampling from a normal distribution to obtain possible “random effects” based on realistic intra-class correlations of 0.3 for subject, as well as adjective. Furthermore, in each run, the empirical Cohen’s f^2^ was extracted to generate a classifiable measure of the effect size. This happened by subtracting the R^2^ from a model without the three-way interaction from the R^2^ of a reduced model with all two-way interactions, but without the three-way interaction. This value was then normalized via the complement of the R^2^ of the full model (see Selya, Rose, Dierker, Hedeker & Mermelstei, 2012).

Supplemental figure 6 shows the power curves for the three-way interaction term. Indeed, the analysis is quite efficient in terms of also detecting small effects (~f^2^ = 0.02 at a regression coefficient for the three-way interaction of 0.1). This could result from the amount of information in the model itself, as each participant is exposed to 16 (adjectives) * 12 (avatars with different BMI) = 192 trials.

We did the same for the adjustment task, this time testing for the two-way interaction between valence and group (vs. a model without any interaction term), also providing different realistic regression coefficients. Supplemental figure 7 shows that the analysis is able to capture quite small effect sizes of f^2^ = 0.02 at 80% detection rate already with about 10 to 15 participants per group. This appears to be a result of the fact that there are 16 adjectives presented to each participant.

For the ANOVA reported concerning the BPI, we simply used G*Power (Faul, Erdfelder, Lang & Buchner, 2007). Here, and probably due to the nature of the analysis with three groups and no within-subjects information, a possible effect size needs to be larger to be detectable. With f^2^ = 0.35, the sample size would need to amount to ~84 to yield a power of .80.


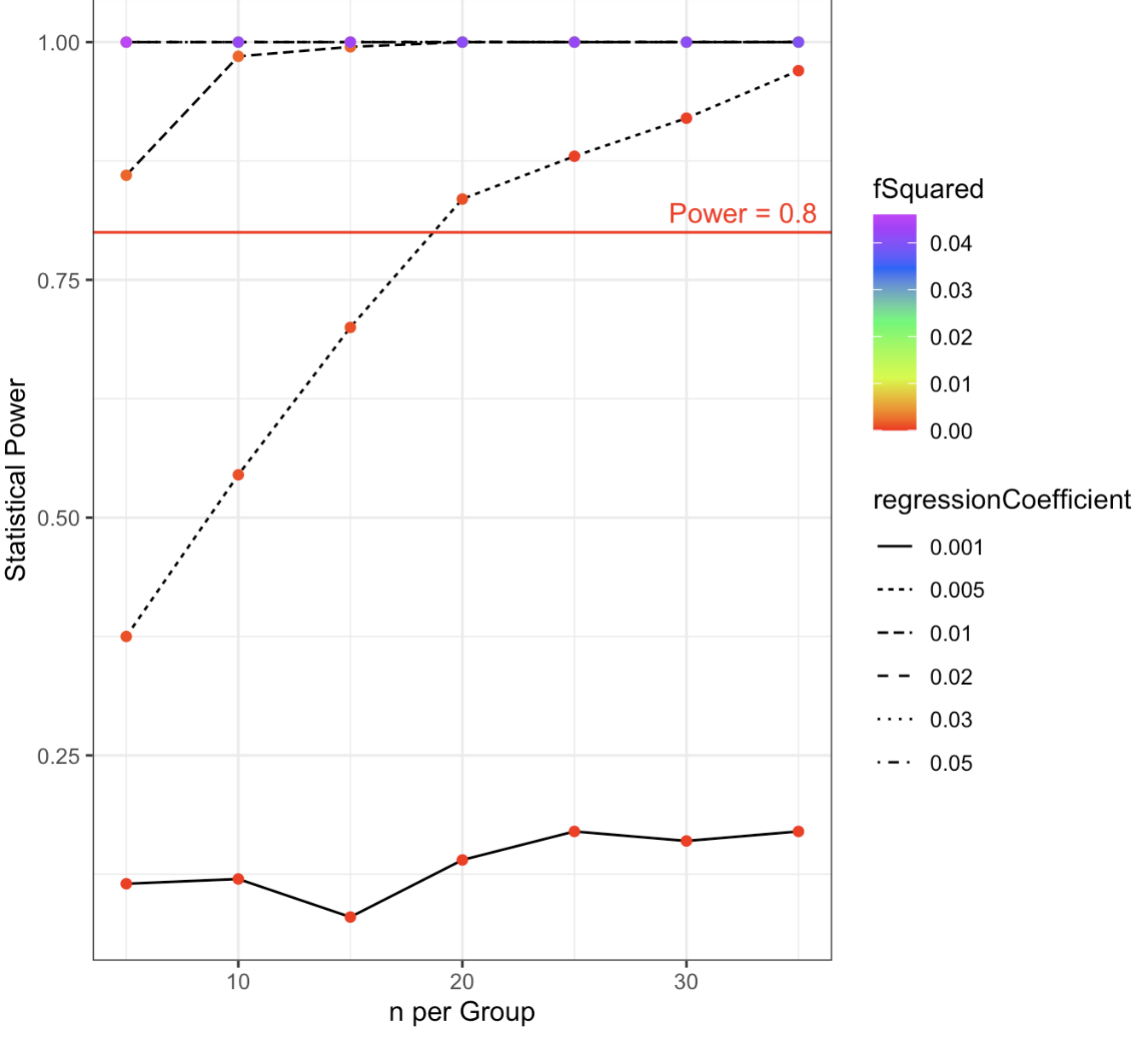


*Supplemental Figure 6.* Power curves for the rating task.


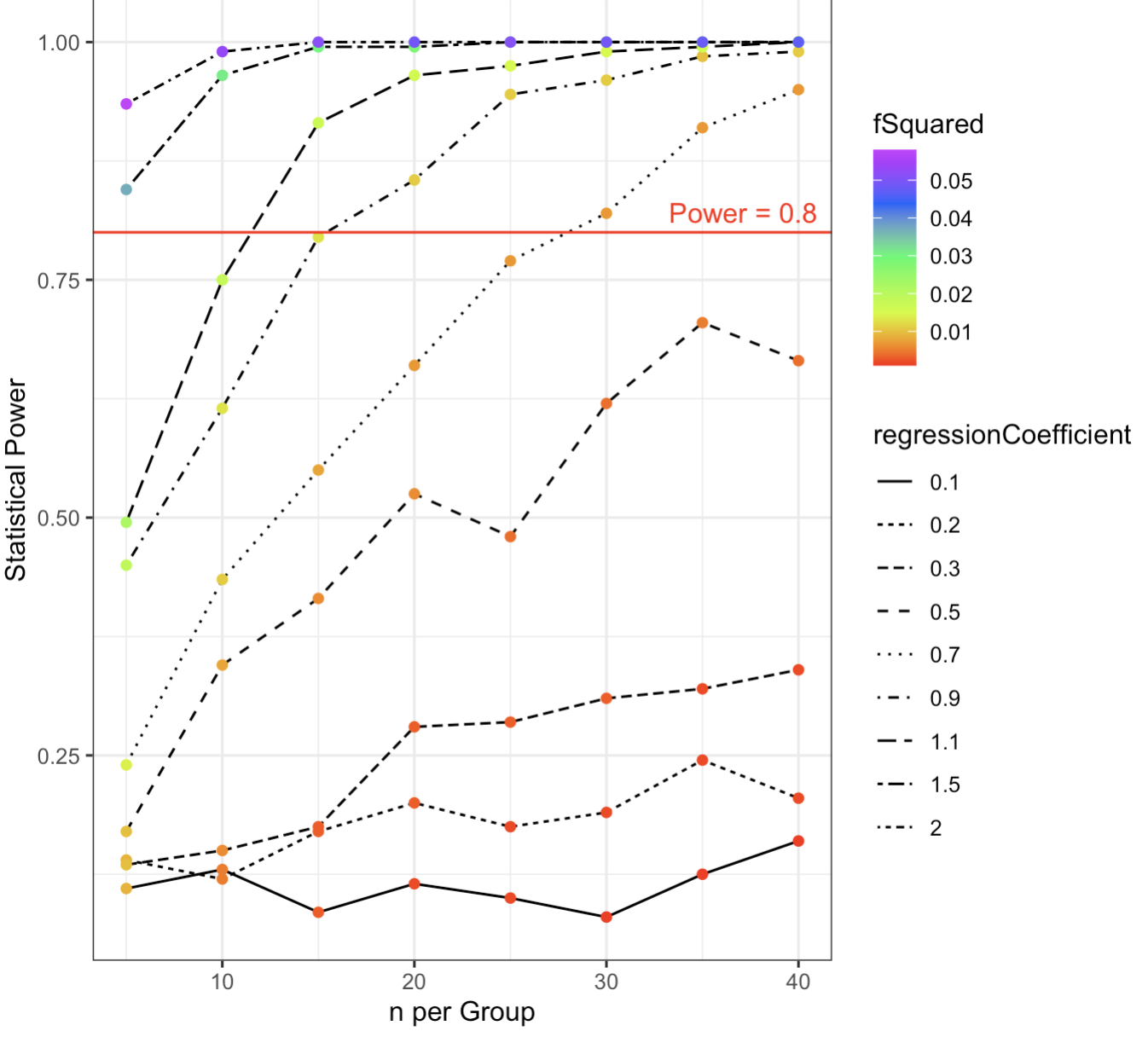


*Supplemental figure 7***.** Power curves for the adjustment task.
